# Profiling of immune related genes silenced in EBV-positive gastric carcinoma identified novel restriction factors of human gammaherpesviruses

**DOI:** 10.1371/journal.ppat.1008778

**Published:** 2020-08-25

**Authors:** Guillaume N. Fiches, Dawei Zhou, Weili Kong, Ayan Biswas, Elshafa H. Ahmed, Robert A. Baiocchi, Jian Zhu, Netty Santoso

**Affiliations:** 1 Department of Pathology, Ohio State University College of Medicine, Columbus, Ohio, United States of America; 2 Gladstone Institute of Virology and Immunology, University of California, San Francisco, California, United States of America; 3 Division of Hematology, Department of Internal Medicine, Ohio State University College of Medicine, Columbus, Ohio, United States of America; University of Southern California, UNITED STATES

## Abstract

EBV-associated gastric cancer (EBVaGC) is characterized by high frequency of DNA methylation. In this study, we investigated how epigenetic alteration of host genome contributes to pathogenesis of EBVaGC through the analysis of transcriptomic and epigenomic datasets from NIH TCGA (The Cancer Genome Atlas) consortium. We identified that immune related genes (IRGs) is a group of host genes preferentially silenced in EBV-positive gastric cancers through DNA hypermethylation. Further functional characterizations of selected IRGs reveal their novel antiviral activity against not only EBV but also KSHV. In particular, we showed that metallothionein-1 (MT1) and homeobox A (HOXA) gene clusters are down-regulated via EBV-driven DNA hypermethylation. Several MT1 isoforms suppress EBV lytic replication and release of progeny virions as well as KSHV lytic reactivation, suggesting functional redundancy of these genes. In addition, single HOXA10 isoform exerts antiviral activity against both EBV and KSHV. We also confirmed the antiviral effect of other dysregulated IRGs, such as IRAK2 and MAL, in scenario of EBV and KSHV lytic reactivation. Collectively, our results demonstrated that epigenetic silencing of IRGs is a viral strategy to escape immune surveillance and promote viral propagation, which is overall beneficial to viral oncogenesis of human gamma-herpesviruses (EBV and KSHV), considering that these IRGs possess antiviral activities against these oncoviruses.

## Author summary

Epstein-Barr virus (EBV), one of the human gamma-herpesviruses, is a well-defined viral agent that strongly associates with malignancies of lymphoid and epithelial origin. EBV-associated gastric carcinoma (EBVaGC) is the most common malignancy caused by EBV infection. In this paper, we identified that beyond tumor suppressor genes, immune related genes (IRGs) are another group of host genes that are extensively DNA hypermethylated and epigenetically silenced in EBVaGC comparing to non-EBV GC. For certain IRGs, such as metallothionein-1 (MT1) and homeobox A (HOXA) genes, the whole gene clusters undergo DNA hypermethylation in EBVaGC, while it also occurs for isolated individual IRGs, such as IRAK2 and MAL. Considering the critical role of innate immunity in antiviral control, we expected that silencing of IRGs would benefit EBV viral replication and propagation. Indeed, our further investigation of several IRGs (MT1G, HOXA10, IRAK2, and MAL) confirmed their antiviral activities against EBV lytic replication. Some of them also suppress lytic replication of another human gamma-herpesvirus, Kaposi’s sarcoma-associated herpesvirus (KSHV), indicating their broad antiviral spectrum. Collectively, our results demonstrated that epigenetic silencing of antiviral IRGs is an efficient viral strategy utilized by oncoviruses, such as EBV, to escape immune surveillance and promote viral propagation, which is overall beneficial to viral oncogenesis.

## Introduction

Gastric cancer (GC) is the fifth most commonly diagnosed cancer worldwide and the third leading cause of cancer-related death with an estimated 783,000 deaths in 2018 [1]. The Cancer Genome Atlas (TCGA) has classified GC into four subtypes [2]: genomically stable tumors (GS), tumors with chromosomal instability (CIN), microsatellite instable tumors (MSI), as well as Epstein-Barr Virus-associated GC (EBVaGC) that accounts for around 10% of all GC [3]. EBVaGC has several distinct characteristics [2] that include: frequent PI3KCA mutations [4,5], altered DNA methylation pattern, amplification of PD-L1 [6,7]. Additionally, EBVaGC has been shown to arise from monoclonal amplification of EBV-infected gastric cells, thus suggesting that viral infection is the early step of carcinogenesis [8,9].

Epstein-Barr virus (EBV), the etiological agent of EBVaGC, is a widespread oncogenic virus that exists in more than 90% of adult population. Early event of EBV infection is poorly understood despite it is known that the virus is transmitted through the saliva [10]. Studies with infectious mononucleosis patients have suggested that the earliest event following primary infection is the expression of lytic genes. Afterward, EBV switches to a latent phase, during which the viral genome remains as an episome largely quiescent in memory B cells with expression of certain viral miRNAs and latent proteins (reviewed in [11]). Occasional viral reactivation may occur within these lymphocytes causing infection of neighboring naïve B cells and viral shedding. The lytic switch from latency is initiated with the expression of immediate early lytic genes, Zta and Rta, which transactivate themselves and early lytic genes, leading to viral DNA amplification [12–14]. Subsequently, late lytic genes encoding glycoproteins as well as tegument and capsid proteins are expressed, and new viral particles are formed [15,16].

In immuno-competent host, infection with EBV induces both innate and adaptive immune responses that subdue the virus and bring the infection under control. EBV lytic and latent proteins are rich in antigens that lead to activation of natural killer cells as well as mobilization of specific CD4^+^ and CD8^+^ T-cells, as anti-EBV immunity. Upon resolution of primary infection, a small pool of memory T-cells is then maintained for continuous immuno-surveillance, which is important for long-term viral control [17]. The fact that EBV successfully establishes lifelong persistence despite host immune responses indicates that EBV has developed a powerful strategy to evade host immuno-surveillance.

Epigenetic alteration of the host genome has emerged as one critical mechanism for subverting immune detection and driving carcinogenesis for oncoviral infection [18]. EBV infection in gastric cancer is associated with genome-wide DNA hypermethylation of the host CpG Islands [19,20]. Although an earlier study linked EBV LMP2A protein to STAT3 phosphorylation and increased DNMT1 level and the aberrant CpG methylation of some host promoters [21], the detailed mechanisms involved remain far from elucidated. Such aberrant modulation in EBVaGC has been linked to downregulation of some tumor suppressor genes [22,23], however less is known about the role of such epigenetic alteration in the context of immune response to EBV infection. In this study we analyze the transcriptomic signatures and methylation profiles of immune-related gene (IRG) to obtain new insights into viral-host interaction in EBVaGC. Subsequent functional characterizations demonstrate the antiviral potency of the deregulated IRGs against EBV and its highly-related gammaherpesvirus, KSHV. This analysis constitutes a new methodology to identify restriction factors to tumor viruses.

## Results

### Deregulation of IRG in EBV-associated gastric cancer (EBVaGC)

**Fig 1:**
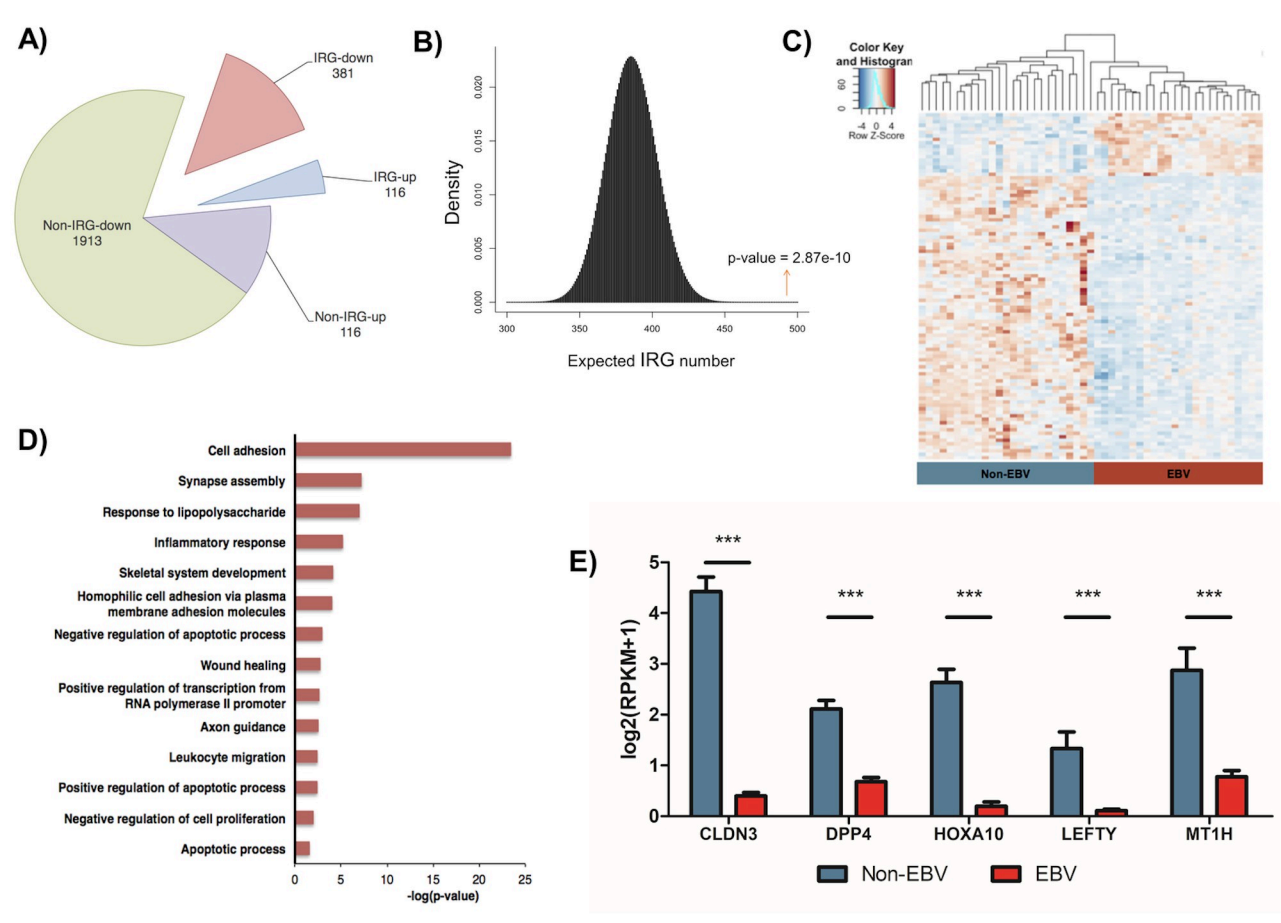
Transcriptomic profiling of immune response genes (IRGs) in EBV-associated gastric cancer (EBVaGC). (A) Pie chart showed dysregulation of IRGs that made up around 20% of all the differentially-expressed (DE) genes in EBVaGC. (B) Hypergeometric simulation plot demonstrated the significant enrichment of IRGs in EBVaGC. The red arrow indicates the identified number of differentially regulated IRGs (497) with the corresponding p value (2.87e-10). (C) Heat map showed the hierarchical clustering of all DE IRGs, comparing between EBV-positive (right, red) and EBV-negative (left, blue) GC samples. (D) Functional enrichment analysis revealed the top biological processes associated with the suppressed IRGs. (E) Normalized mRNA expression of selected top down-regulated IRGs was compared between EBVaGC and non-EBV GC samples. Results were based on n = 23 and 25 samples, for EBVaGC and non-EBV respectively, and presented as mean ± SEM., (* p<0.05; ** p<0.01; *** p<0.001, Wilcoxon signed-rank test).

We analyzed the transcriptome of EBV-positive and negative gastric cancers using the TCGA RNA-seq data. A total of 2,723 host genes were found to be differentially expressed: 2294 genes were down-regulated while 429 genes were up-regulated (**Fig 1A**). We defined these differentially expressed (DE) genes with two following criteria: fold change above 2 and p-value lower than 0.05. Given the critical role of IRGs in regulating both viral infections and cancer developments, we particularly focused on this group of genes for further analysis. A curated list of various published IRGs (4215 genes) was manually compiled to serve as a reference (**S1 Table**). We identified that 381 IRGs were down-regulated while 116 IRGs were up-regulated (**Fig 1A, S2 Table**), which in total accounts for almost twenty percent of all DE genes in EBVaGC. Hypergeometric analysis confirmed that such enrichment of IRGs as the DE genes was statistically significant (**Fig 1B**, p-value = 2.87e-10). Non-supervised hierarchical clustering further distinguished the DE IRG into two subtypes: the EBV-negative and -positive cancers (**Fig 1C**). The EBV-positive gastric cancer subtype showed predominant downregulation of IRGs (over 75%) with only a small portion of IRGs were upregulated whereas the EBV-negative samples showed the reverse phenotypes **(Fig 1C**).

To gain more information about cellular functions and pathways that are affected by deregulation of IRGs, we performed functional enrichment analysis of all the DE IRGs. Down-regulated IRGs identified in EBVaGC were involved in a wide range of cellular functions, such as cellular adhesion, synapse assembly, response to lipopolysaccharides, wound healing, and apoptosis (**Fig 1D**). Transcriptional profiles of certain top down-regulated IRGs in EBV+/- GCs, such as CLDN3, DPP4, HOXA10, LEFTY1, and MT1H were illustrated (**Fig 1E**). Consistent with our finding, CLDN3 was previously reported to express at a reduced level in EBVaGC [24,25], while DPP4 is an androgen receptor-induced tumor suppressor gene that was downregulated in some cases of prostate cancer. Similarly, HOXA10, LEFTY1, and MT1H have all been suggested to act as tumor suppressors, whose overexpression leads to a better prognosis [25–28].

On the contrary, the up-regulated IRGs in EBVaGC were mostly involved in immune response, such as interferon gamma signaling pathway, T-cell receptor signaling pathway, adaptive immune response, and inflammatory response (**S1A Fig**). It is interesting to note that certain level of inflammatory response can favor tumorigenesis [29], which leads us to speculate that EBV may elevate inflammation to a certain level that benefits EBV oncogenesis. We also observed enrichment of genes involved in cytolysis process, which is usually caused by production of progeny viruses [30]. It is likely due to modulation of certain IRGs that could benefit viral replication of EBV. The transcriptional profile of certain top up-regulated IRGs was shown as well (**S1B Fig**). It has been suggested that EBV lytic replication is also critical for cellular transformation through the release of certain growth factors [31,32]. In fact, we indeed observed expression of EBV lytic genes in RNA samples of EBVaGC (**S1C Fig).** Therefore, it is possible that EBV lytic replication may contribute to gastric carcinogenesis beyond the mere purpose of viral propagation and spreading.

### Methylation profile of IRG in EBVaGC

One of EBVaGC defining features is host DNA hypermethylation [19,20], thus we next investigated whether dysregulation of IRG expression in EBVaGC was mostly due to this epigenetic regulation. We analyzed the DNA methylation status of host genes in EBV-positive and negative gastric cancers using the TCGA 450K CpG array data. We categorized the differentially methylated (DM) genes with the criteria that two or more probes for each gene are differentially methylated with p-value <0.05. Among all the DM genes, we particularly focused on the DE IRGs to determine the correlation of their DNA methylation status with their expression level.

**Fig 2:**
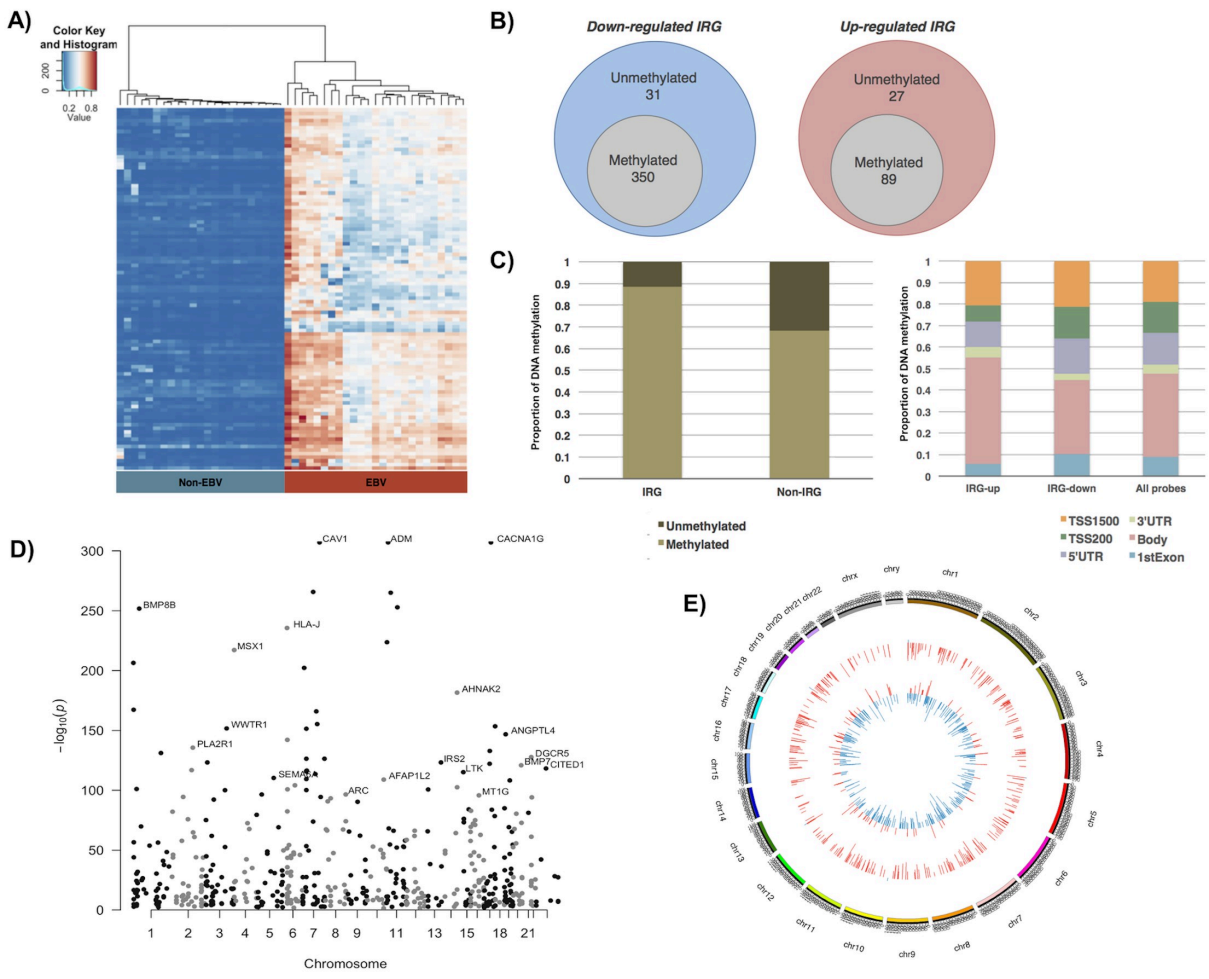
DNA methylation analysis of IRGs in EBVaGC. (A) Heat map showed the hierarchical clustering of all differentially methylated (DE) IRGs, comparing between EBV-positive (right, red) and EBV-negative (left, blue) GC samples. (B) Venn diagram showed the number of DE IRGs (grey circle) for both down-regulated IRGs (left, blue circle) and up-regulated IRGs (right, red circle). (C) (Left panel) Bar graph showed the proportion of methylated and unmethylated genes in deregulated IRG and non-IRG groups. (Right panel) Stacked bar graph showed the distribution of methylated CpG probes at different regions, including TSS1500 (orange), TSS200 (green), 5’UTR (purple), 3’UTR (light green), gene body (pink), and 1^st^ Exon (blue), for up-regulated and down-regulated IRGs in EBVaGC. TSS200: 200bp upstream of transcription start site (TSS); TSS1500: 200-1500bp upstream of TSS. Proportion of each regions in the whole array is included. (D) Manhattan plot showed the chromosomal localization (x-axis) and log-base of adjusted p-value (y-axis) of all differentially methylated IRGs in EBVaGC. Examples of top methylated IRGs were annotated. (E) Circos diagram of all IRGs in EBVaGC depicts the methylation pattern in the outer circle (hypomethylated or hypermethylated gene position is displayed as blue or red respectively), and the transcriptional pattern in the inner circle (down-regulated or up-regulated gene position is displayed as blue or red respectively).

As expected, our analysis confirmed that most of down-regulated IRGs in EBVaGC had undergone DNA hypermethylation (**S2 Table**). Unsupervised hierarchical clustering was performed for the top 100 DM IRGs between EBV-negative and positive cancers, showing their DNA hypermethylation pattern in almost all genes involved (**Fig 2A**). In total, we identified 350 genes that were hypermethylated within 381 downregulated IRGs (**Fig 2B**, left panel). Therefore, more than 91% of down-regulated IRGs were methylated, which was higher than non-IRGs with only 72% of down-regulated genes are methylated **(S2A Fig)**. Overall, we saw a twenty percent increase of DNA methylation in IRG loci (88%) when compared to non-IRG sites (68%) **(Fig 2C,** left panel, p-value < 0.0001, Chi-square test), indicating that that IRG had higher tendency to be dysregulated through DNA hypermethylation in EBVaGC.

Interestingly, we also found that among 116 up-regulated IRGs, 89 of them were hypermethylated (**Fig 2B**, right panel), indicating their unique epigenetic regulation. DNA methylation sites determine the outcome of gene transcription. In general, it is believed that DNA methylations occurring at promoter regions silence gene expression while DNA methylations occurring at gene bodies promote gene expression [33–35].

We examined the methylation loci with respect to the dysregulated IRGs. We found that the CpG Island, predominantly located within promoter regions, were the primary methylation sites of the down-regulated IRGs (as high as 42%), whereas the up-regulated IRGs or all the probes displayed much lower proportions with 24.2% and 32% of CpG Island methylation respectively **(S2B Fig,** p-value < 0.0001, Chi-square test). On the other hand, the up-regulated IRGs had the highest methylation at the Open Sea region (37.4%) compared to the down-regulated IRGs (27%) or the average rate of all the probes (35.1%) (**S2B Fig**). Open sea region is located the furthest from the CpG Island with more than 4 kb away. Further investigation revealed that 52.4% of methylation occurred within the promoter region (TSS1500 + TSS200 + 5’UTR) of down-regulated IRGs, while up-regulated IRGs and all CpG probes had 39.7% and 47.9% promoter methylation respectively (**Fig 2C, right panel,** p-value < 0.05, Chi-square test), and that 34.5% of methylation occurred within the gene body of down-regulated IRGs, while up-regulated IRGs and all CpG probes had 49.4% and 38.6% gene body methylation respectively (p-value < 0.0001). Our results indicated that IRGs were substantially subjected to EBV-induced DNA hypermethylation, and that their down- or up-regulation highly depends on the preferred sites for DNA methylation either at promoter region or gene body. In addition, other epigenetic mechanisms may also complicate the outcome of IRG gene expression beyond DNA methylation.

Some of the top DNA hypermethylated IRGs, such as BMP8B, PLA2R1, MSX1, DGCR5, and MT1G, were previously identified as tumor suppressors in gastric and other cancers [36–40] (**Fig 2D**). As a summary, we illustrated the overall transcriptomic and methylation patterns of IRGs with circular Circos diagram (**Fig 2E**), showing the concordance of DNA hypermethylation pattern (outer ring, red bar) with the down-regulated expression of IRGs (inner ring, blue bar). These results concluded that EBV-induced DNA hypermethylation mainly leads to the silencing of IRGs, which were subjected to further functional characterization.

### Epigenetic silencing of MT1 and HOXA gene clusters in EBVaGC

From the analysis of IRG transcriptome and DNA methylome associated with EBVaGC, we noticed that certain down-regulated IRGs were isoforms/homologs accumulating as the gene clusters undergoing massive DNA hypermethylation, such as the metallothionein-1 (MT1) and homeobox A (HOXA) gene clusters. MT1 is a subfamily of metallothionein protein that primarily function to protect cell from heavy metal toxicity and oxidative stress. MT1 proteins include several different subtypes (A, B, E-H, L, M, X) that are located at the chromosome 16 as a gene cluster [41]. Likewise, the HOXA gene cluster, locating at the chromosome 7p15.2, encodes HOXA1-11, 13 proteins that mainly function as transcription factors during developmental process [42]. Previous studies have linked the down-regulation of MT1F, MT1G, and MT1H to hepatocellular carcinoma [28,40,43], and the down-regulation HOXA to breast cancer with the whole cluster silenced [44]. In relevance to viral infection, MT1F, MT1G, and MT1H proteins exerted some amount of antiviral activity against Hepatitis C viruses (HCV) [45,46], while HOXA10 was shown to suppress Hepatitis B viruses (HBV) replication [42].

**Fig 3:**
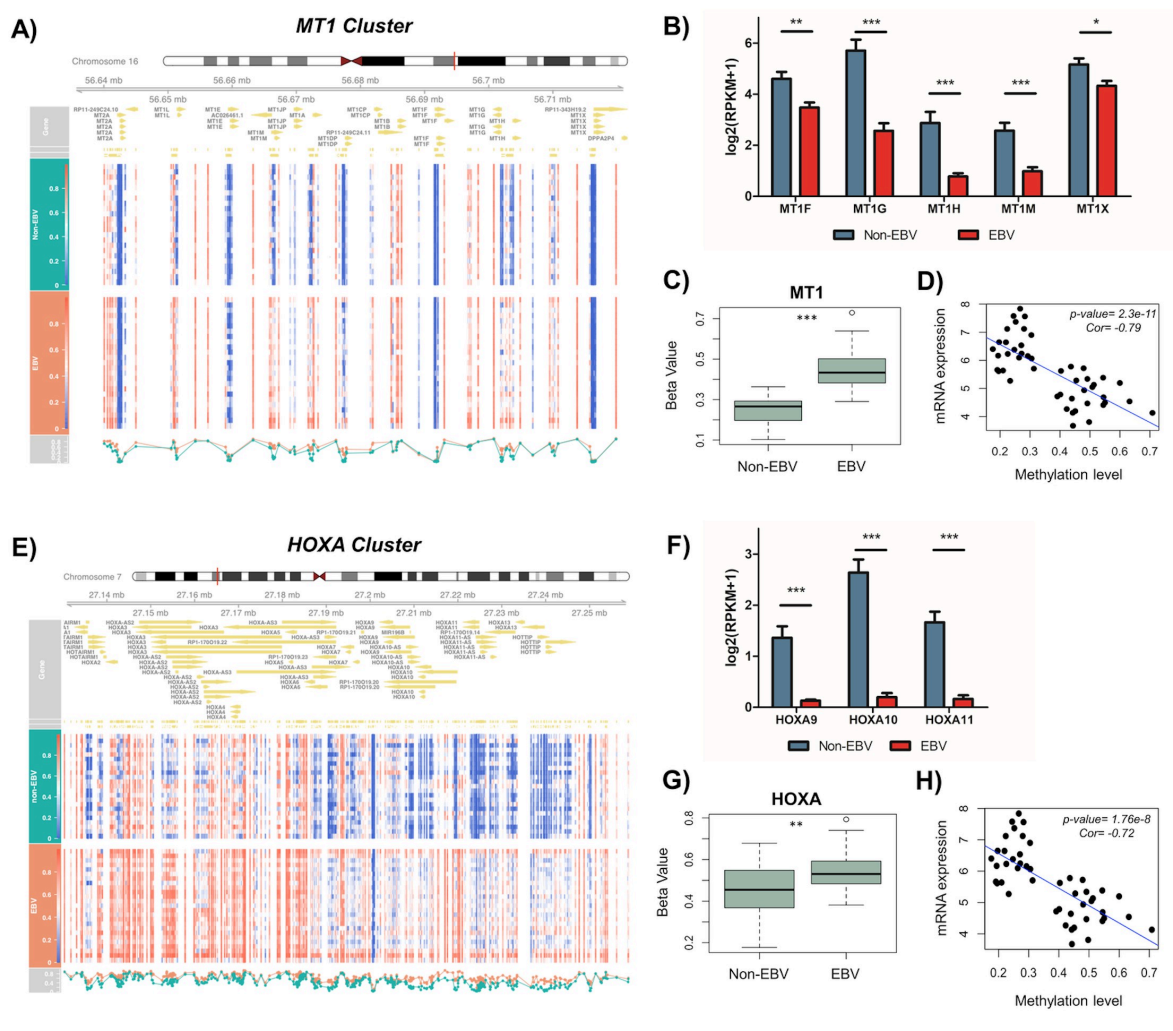
DNA hypermethylation and down-regulation of MT1 and HOXA gene clusters in EBVaGC. (A, E) Heat map showed the beta value of each CpG probe located within MT1 (A) and HOXA (E) gene clusters. EBV-negative GC samples (n = 25), upper panel; EBVaGC samples (n = 23), lower panel. (B, F) Normalized mRNA expression of MT1 (B) and HOXA (F) genes was compared between EBVaGC and non-EBV GC samples. Results were based on n = 23 and 25 samples, for EBVaGC and non-EBV respectively, and presented as mean ± SEM, (* p<0.05; ** p<0.01; *** p<0.001, Wilcoxon signed-rank test). (C, G) Box plot showed the distribution of beta values for all CpG probes located within MT1 (C) and HOXA (G) gene cluster in EBVaGC and non-EBV GC samples. Results were based on n = 23 (EBV +) and 25 (EBV -) samples and presented as mean ± S.D. (* p<0.05; ** p<0.01; *** p<0.001, Wilcoxon signed-rank test). (D, H) Correlation plot showed the inverse relationship between mRNA expression and methylation beta values for MT1 (D) and HOXA (H) gene clusters. P-value and Pearson’s correlation coefficient are indicated.

Our analysis showed that DNA hypermethylation occurs almost across the entire MT1 gene cluster (**Fig 3A**), and expression of its posterior genes (MT1F, G, H, M, and X) were significantly reduced in EBVaGC compared to non-EBV GC (**Fig 3B**). Moreover, beta values of MT1 gene cluster, indicating the overall methylation level, were higher in EBVaGC compared to non-EBV GC (**Fig 3C**). We further saw a significant inverse relationship between DNA methylation and expression level of MT1 genes (**Fig 3D**) confirming that DNA hypermethylation indeed contributes to the gene repression of MT1 gene cluster. Similarly, we observed accumulation of DNA hypermethylation in the HOXA gene cluster in EBVaGC, as evidenced by the overall distribution pattern (**Fig 3E**) as well as the higher beta values in EBVaGC vs non-EBV GC (**Fig 3G**). Consistently, expression of HOXA9, HOXA10, and HOXA11 were strongly reduced in EBVaGC compared to non-EBV GC (**Fig 3F**). The Pearson’s correlation analysis also showed a significant negative association between DNA methylation and expression level of HOXA genes within the same gene cluster (**Fig 3H**). Overall, our results demonstrated that the global aberrant DNA methylation pattern observed in EBVaGC results in suppression of an entire gene cluster, which could form an epigenetic microdeletion to efficiently silence host genes unfavorable for viral replication and oncogenesis. This could be an effective viral strategy to not only generate long-lasting pro-viral and/or tumor-promoting effects but also prevent functional compensation by targeting homolog genes within the same gene cluster.

### Down-regulation of MT1 and HOXA genes in EBV-positive cell lines

**Fig 4:**
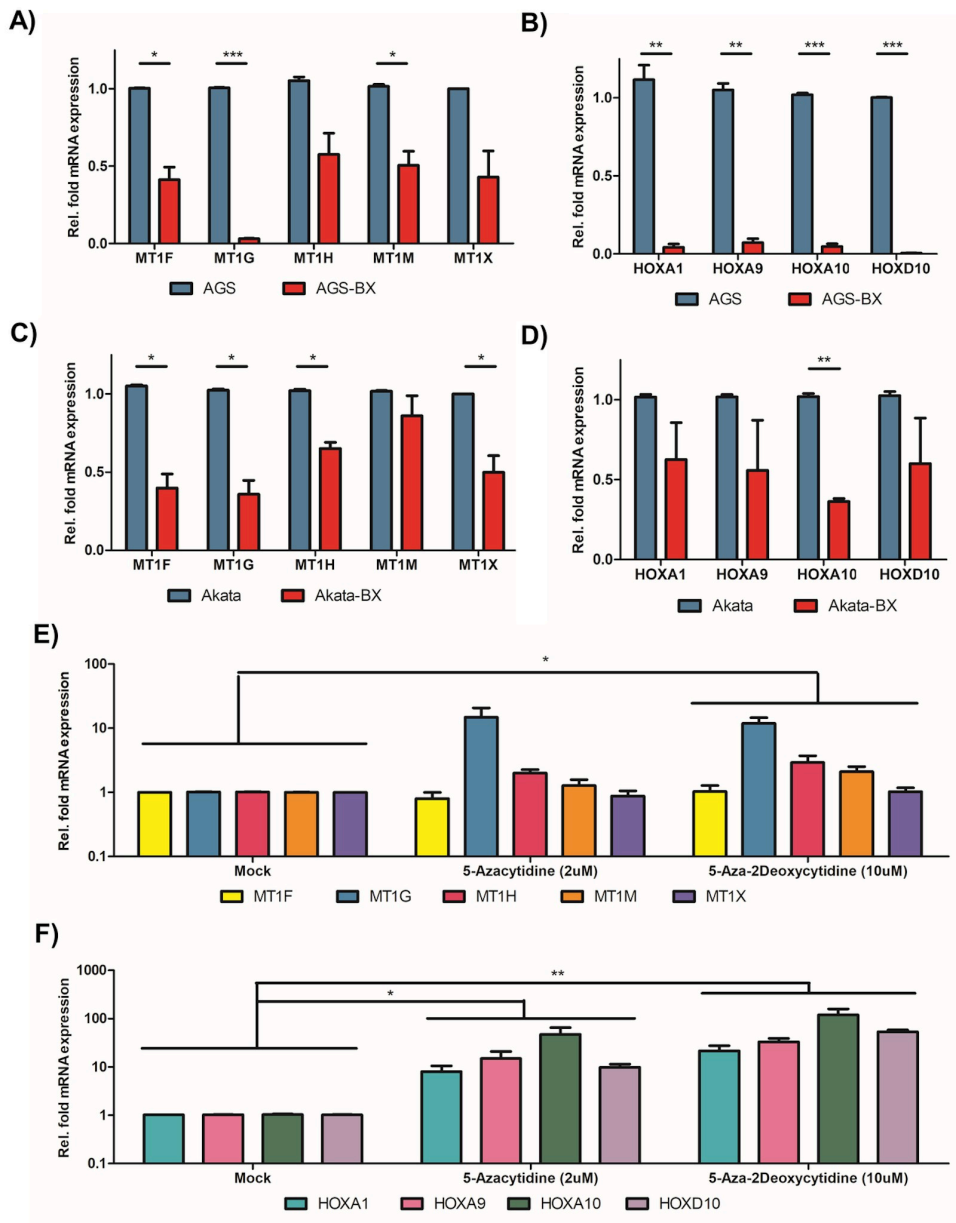
Down-regulation of MT1 and HOXA genes in EBV-positive GC and Burkitt’s lymphoma cell lines. (A, B) AGS-BX and AGS gastric cancer cells were subjected to RT-qPCR assays to measure the expression of MT1 (A) and HOXA (B) genes. Relative expression in AGS-BX (Red) of each gene was normalized with respect to parental AGS cells (Blue). (C, D) Akata-BX and Akata Burkitt's lymphoma cells were assayed similarly as (A, B) to measure the expression of MT1 (C) and HOXA (D) genes. Relative expression in Akata-BX (Red) of each gene was normalized with respect to parental Akata cells (Blue). (E, F). AGS-BX cells were treated with DNA methylation inhibitors (5-azacytine, 5-aza-2-deoxycytine), or vehicle control, followed by the RT-qPCR assays to measure the expression of selected MT1 (E) and HOXA (F) genes. Results are based on n = 3 independent repeats and presented as mean ± SEM. (* p<0.05; ** p<0.01; *** p<0.001, two-tailed paired Student t-test).

In order to confirm whether EBV leads to the down-regulation of MT1 and HOXA genes, we next measured the expression level of these genes by reverse transcription coupled quantitative real-time PCR (RT-qPCR) in the isogenic human gastric adenocarcinoma cell lines of epithelial origin either harboring EBV (AGS-BX) or not (AGS). The BX recombinant EBV strain bears two open-reading frames (the G418 resistance and GFP genes) within the non-essential viral gene BXLF1 of EBV Akata strain [47]. Our results showed that for all the deregulated MT1 genes found in EBVaGC patients (MT1F, MT1G, MT1H, MT1M, and MT1X) as well as some representative HOXA genes (HOXA1, HOXA9, HOXA10) with an additional gene from the HOXD gene cluster (HOXD10) were all down-regulated in AGS-BX cells compared to AGS cells. The reduction of MT1G, in particular, was striking and almost abolished the gene expression (> 95% reduction), while the reduction of other MT1 genes were significant but not as strong (**Fig 4A**). For HOXA genes, we found that all of them had more than 90% of reduced expression in AGS-BX *vs* AGS cells (**Fig 4B**).

To determine whether these observations were driven by EBV or the cancer type, we also measured expression of the same MT1 and HOX genes in another two isogenic Burkitt’s lymphoma (BL) cell lines with or without EBV (Akata-BX vs Akata) [48]. We found that these genes were also down-regulated in Akata-BX cells when compared to Akata cells (**Fig 4C and 4D**) similar to the results in gastric cancer cell lines although to a lesser degree. Interestingly, we found that HOXA10 had the most reduction in lymphoma cell lines, which was similar to our observation from the EBVaGC clinical samples (**Fig 3F**). These results suggest that EBV infection induces down-regulation of MT1 and HOXA genes regardless of the type of cancer.

To verify that EBV-mediated down-regulation of these IRGs was through DNA methylation, we treated the AGS-BX cells with two DNA methylation inhibitors, 5-Azacytidine and 5-Aza-2’-deoxycytidine [49]. Expression of MT1 genes (MT1F, MT1G, MT1H, MT1M, and MT1X) (**Fig 4E**) as well as HOX genes (HOXA1, HOXA9, HOXA10, HOXD10) (**Fig 4F**) were determined by RT-qPCR assays. Expression of MT1G was significantly up-regulated in AGS-BX cells treated with both DNA methylation inhibitors whereas the other MT1 genes were only moderately up-regulated, in concordance with the RT-qPCR pattern of MT1 genes in AGS-BX vs AGS cells (**Fig 4A**). Similarly, expression of HOX genes (HOXA1, HOXA9, HOXA10, HOXD10) were significantly up-regulated in AGS-BX cells treated with both DNA methylation inhibitors, also in concordance with the RT-qPCR pattern of HOX genes in AGS-BX vs AGS cells (**Fig 4B**). Cytotoxicity of DNA methylation inhibitors in AGS-BX cells was measured, which was minimal (**S3A Fig**). Furthermore, we measured the CpG methylation level of HOXA10 and MT1G promoter regions covering hypermethylation loci identified from EBVaGC patients (TCGA 450k CpG methylation array) in AGS-BX and AGS cells by using the Methylated DNA Immunoprecipitation (MeDIP) coupled with qPCR (MeDIP-qPCR) assay. The results showed that both HOXA10 and MT1G promoter regions possess higher level of CpG methylation in AGS-BX cells in comparison with AGS cells (**S3B Fig**).

These results using the simplified cancer cell models of EBV infection were consistent with those obtained *in vivo* from patient specimens. Taken together, we have confirmed that EBV infection drives DNA hypermethylation, leading to epigenetic silencing of IRG, such as MT1 and HOXA gene cluster. It has been previously reported that latent EBV induces DNA hypermethylation [19,20,50], and that EBV-mediated silencing of tumor suppressor genes, such as IHH and TRABD, favors tumor proliferation [22]. However, such regulation of IRG by EBV was not previously investigated. We postulate that epigenetic silencing of IRG favors viral replication and spreading given to the antiviral nature of host immune responses. Through this avenue, we would be able to identify novel IRG against EBV.

### Identification of MT1 genes as antiviral factors against EBV

**Fig 5:**
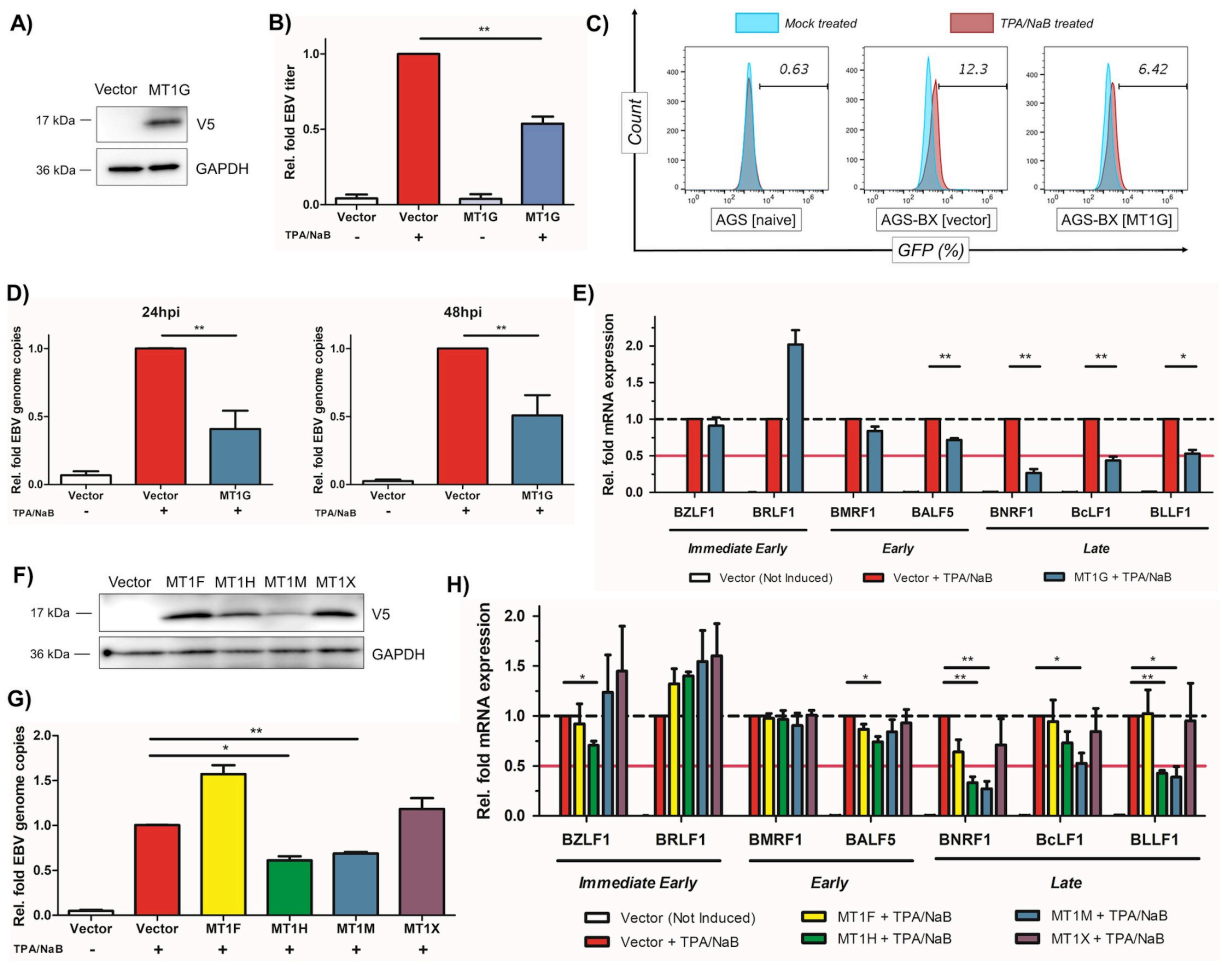
MT1G and other MT1 genes suppress lytic replication of EBV. (A) Protein expression of V5-tagged MT1G in stably transduced AGS-BX cells was verified by immunoblotting. (B) Newly produced EBV viruses from TPA/NaB treated or untreated AGS-BX cells stably expressing V5-MT1G or the vector control were titrated using Raji cells via flow cytometry. (C) Representative diagram of flow cytometry for (B). AGS cells were used to determine the background. (D) EBV genome copies (EBNA1 DNAs) in TPA/NaB treated AGS-BX cells stably expressing V5-MT1G or the vector control in the presence of Zinc (50uM) were measured by qPCR assays at 24 hpi (left panel) and 48 hpi (right panel). The relative level of EBNA1 DNAs in TPA/NaB treated AGS-BX cells transduced with the vector control was set up as 1. (E) Expression of EBV lytic genes (BZLF1, BRLF1, BMRF1, BALF5, BNRF1, BcLF1, BLLF1) in TPA/NaB treated or untreated AGS-BX cells stably expressing V5-MT1G or the vector control in the presence of Zinc (50uM) were measured by RT-qPCR assays at 24hpi. (F) Protein expression of V5-tagged MT1 genes (MT1F, H, M, X) in stably transduced AGS-BX cells was verified by immunoblotting. (G) EBV genome copies in TPA/NaB treated AGS-BX cells stably expressing V5-tagged MT1 genes (MT1F, H, M, X) or the vector control in the presence of Zinc (50uM) were measured by qPCR assays at 24hpi, similar as (D). (H) Expression of EBV lytic genes in TPA/NaB treated or untreated AGS-BX cells stably expressing V5-tagged MT1 genes (MT1F, H, M, X) or the vector control in the presence of Zinc (50uM) were measured by RT-qPCR assays at 24hpi, similar as (E). All of above results are based on n = 3 independent repeats and presented as mean ± SEM (* p<0.05; ** p<0.01; *** p<0.001, two-tailed paired Student t-test).

MT1 proteins are crucial for antimicrobial defense due to their role in cellular protection from reactive oxygen species (ROS). ROS itself is involved in host defense of pathogens infection and an earlier study has shown that several MT1 proteins possess mild anti-viral activity against HCV [45]. However, it has never been examined whether MT1 proteins also inhibit EBV. We initially tested the impact of MT1G on EBV lytic replication, given that MT1G was the most suppressed isoform among tested MT1 genes in AGS-BX cells (**Fig 4A**). We stably expressed a V5-tagged MT1G protein in pLEX vector (pLEX-MT1G) or the empty vector in AGS-BX cells and expression of V5-MT1G was verified by immunoblotting (**Fig 5A**). The stable cells were then subjected to 12-O-Tetradecanoylphorbol 13-acetate and Sodium Butyrate (TPA/NaB) treatment to induce EBV lytic replication and the newly produced infectious EBV particles were quantified using Raji cell infection assay via flow cytometry. Our results showed that overexpression of MT1G in AGS-BX cells resulted in a 50% reduction of EBV titer in comparison to the empty vector (**Fig 5B** and **5C**).

Metallothioneins fold around the metallic cation they bind [51,52], therefore we also added extra Zn^2+^ at a previously reported physiological concentration [45]. We further determined whether MT1G suppressed EBV viral genome amplification and lytic gene expression, thus reducing EBV titer. The qPCR assays using primers targeting the EBNA1 region of EBV genome indicated that overexpression of MT1G caused a significant decrease of EBV genome copies by more than 50% at both 24 and 48 hours post induction (hpi) (**Fig 5D**). The RT-qPCR assays of EBV lytic genes (immediate early, early, and late) also identified that overexpression of MT1G dramatically reduced the expression of EBV late lytic genes (BNRF1, BcLF1 and BLLF1) (**Fig 5E**). Coordinately, we also assessed the MT1G association with promoter region of various EBV lytic genes via chromatin immunoprecipitation (ChIP) coupled with qPCR (ChIP-qPCR) assays. The results showed that MT1G particularly associates with the promoter region of BLLF1, which is further enhanced due to TPA/NaB-induced EBV reactivation (**S4A Fig**). This result was consistent with the MT1G-mediated decrease of EBV genome amplification, as continuous genome replication is critical for expression of EBV late lytic genes [53]. However, expression of EBV early lytic genes (BMRF1 and BALF5) was only marginally decreased in MT1G-expressing AGS-BX cells. Although early lytic genes are primarily dedicated to viral DNA replication, for example BALF5 encoding viral DNA polymerase, such moderate reduction of EBV early gene expression may not cause huge reduction of overall EBV genome.

Metallotiotheins are a large family of duplicated genes showing a high degree of redundancy [54–56]. We determined the anti-EBV activities of other down-regulated MT1 genes. We generated AGS-BX cells stably expressing MT1F, MT1H, MT1M, and MT1X in pLEX vector, whose protein expression was verified by immunoblotting (**Fig 5F**). Using these stable cells, we analyzed both viral genome amplification (**Fig 5G**) and individual lytic genes expression (**Fig 5H**) upon reactivation induction with TPA/NaB. We found that overexpression of MT1H and MT1M, but not MT1F and MT1X, lead to the reduction of EBV genome (**Fig 5G**). Similar to MT1G, overexpression of MT1H and MT1M suppressed expression of EBV late lytic genes (BNRF1, BcLF1, and BLLF1) rather than the earlier genes (**Fig 5H**). As expected, overexpression of MT1F and MT1X showed no obvious inhibitory effects on any of EBV lytic genes (**Fig 5H**). Overall, our results suggest that some members of MT1 protein family, such as MT1G, MT1H, and MT1M, share certain level of functional redundancy and possess the similar antiviral activity against EBV lytic replication. Furthermore, we tested whether MT1 proteins also inhibit lytic replication of Kaposi’s sarcoma-associated herpesvirus (KSHV), another human gammaherpesvirus, in the HEK293.r219 cells, which was previously used for identification of host regulators of KSHV lytic replication [57]. These cells harbor the recombinant KSHV genomes that encode the GFP gene at the downstream of the eF1α promoter and the RFP gene downstream of the KSHV lytic PAN promoter, which indicate the KSHV latent and lytic infections respectively [58]. We identified that MT1H knockdown by its siRNAs enhances the KSHV lytic replication induced by exogenous expression of KSHV viral transactivator ORF50 (also known as Rta) in HEK293.r219 cells (**S5 Fig**). These results suggest that MT1 proteins, with certain redundancies, inhibits the lytic replication of both EBV and KSHV.

### Identification of HOXA10 as an antiviral factor against EBV and KSHV

**Fig 6:**
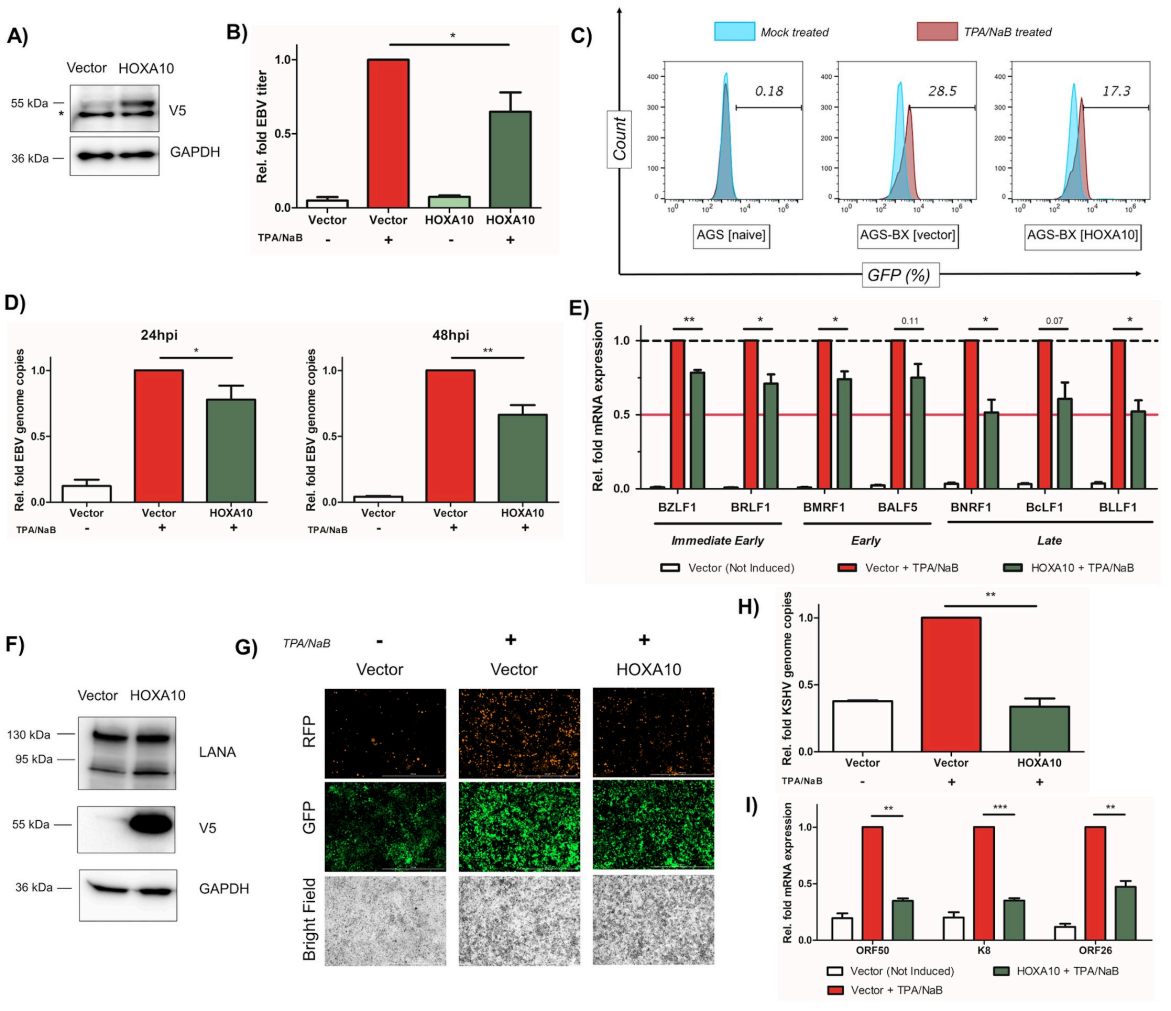
HOXA10 suppresses lytic replication of EBV and KSHV. (A) Protein expression of V5-tagged HOXA10 in stably transduced AGS-BX cells was verified by immunoblotting. * indicates a non-specific protein band. (B) Newly produced EBV viruses from TPA/NaB treated or untreated AGS-BX cells stably expressing V5-HOXA10 or the vector control were titrated using Raji cells via flow cytometry. (C) Representative diagram of flow cytometry for (B). AGS cells were used to determine the background. (D) EBV genome copies (EBNA1 DNAs) in TPA/NaB treated AGS-BX cells stably expressing V5-HOXA10 or the vector control in the presence of Zinc (50uM) were measured by qPCR assays at 24 hpi (left panel) and 48 hpi (right panel). The relative level of EBNA1 DNAs in TPA/NaB treated AGS-BX cells transduced with the vector control was set up as 1. (E) Expression of EBV lytic genes (BZLF1, BRLF1, BMRF1, BALF5, BNRF1, BcLF1, BLLF1) in TPA/NaB treated or untreated AGS-BX cells stably expressing V5-HOXA10 or the vector control were measured by RT-qPCR assays at 24 hpi. (F) Protein expression of V5-tagged HOXA10 in transiently transfected HEK293.r219 cells was verified by immunoblotting. Protein expression of KSHV LANA was also probed. (G) Expression of GFP and RFP proteins in TPA/NaB treated or untreated HEK293.r219 cells transiently transfected with V5-HOXA10 or the vector control was visualized by fluorescence microscopy (scale bar = 1mm). (H) KSHV genome copies (ORF73/LANA DNAs) in TPA/NaB treated HEK293.r219 cells transiently expressing V5-HOXA10 or the vector control were measured by qPCR assays at 24 hpi. (I) Expression of KSHV lytic genes (ORF50/Rta, K8/K-bZIP, and ORF26) in TPA/NaB treated or untreated HEK293.r219 cells transiently expressing V5-HOXA10 or the vector control were measured by RT-qPCR assays at 24hpi. All of above results are based on n = 3 independent repeats and presented as mean ± SEM (* p<0.05; ** p<0.01; *** p<0.001, two-tailed paired Student t-test).

Recently, HOXA10 has been reported as a new antiviral protein inhibiting replication of HBV [59]. We have also identified HOXA10 as the most significantly repressed isoform within the HOXA cluster in EBVaGC patients, gastric cancer and lymphoma cell lines (**Figs 3F, 4B and 4D**), we therefore decided to evaluate the antiviral potential of HOXA10 against EBV. We adopted the same protocol as our earlier investigations with the MT1 cluster. We stably transduced V5-tagged HOXA10 in pLEX vector or the empty vector in AGS-BX cells, and the expression of V5-HOXA10 was verified by immunoblotting (**Fig 6A**). Newly produced EBV particles were measured by Raji cell infection assay after lytic induction of these cells with TPA/NaB.

Overall, we saw a little less than 50% reduction of the EBV titer in HOXA10 cells (**Fig 6B**), through the measurement of GFP-positive Raji cells (**Fig 6C**). We also determined the impact of HOXA10 on EBV genome and lytic genes expression. We found that overexpression of HOXA10 mildly reduced EBV genome amplification in AGS-BX cells treated with TPA/NaB at both 24 and 48 hpi with more pronounced effect observed at 48 hpi (**Fig 6D**). Consistent with our titration result, we also found that overexpression of HOXA10 modestly restricts the overall expression of all tested EBV lytic genes (**Fig 6E**), including immediate early (BZLF1, BRLF1), early (BMRF1, BALF5), and late lytic (BNRF1, BcLF1 and BLLF1) genes. Similar to MT1G, the ChIP-qPCR assays identified that HOXA10 also significantly associates with the promoter region of BLLF1 (**S4B Fig**). Overall, our results suggested that HOXA10 indeed suppresses EBV lytic replication albeit at the moderate level.

Previous study showed that inhibition of HBV by HOXA10 is due to negative regulation of the p38 mitogen-activated protein kinase (MAPK) signaling pathway [59], which was also identified as critical for the lytic replication of gamma-herpesviruses, including both EBV [60,61] and KSHV [62]. Therefore, it is likely that beyond EBV, HOXA10 also suppresses KSHV lytic replication as well. We transiently transfected pLEX-HOXA10 or the empty vector into HEK293.r219 cells, and protein expression of V5-HOXA10 was verified by immunoblotting (**Fig 6F**). Lytic induction of these cells with TPA/NaB, followed by the fluorescence imaging showed an apparent diminution of RFP-positive (KSHV-reactivated) cells suggesting an inhibition of KSHV reactivation (**Fig 6G**). To confirm this phenotype, we determined the impact of HOXA10 on KSHV viral genome amplification and lytic gene expression. Overexpression of HOXA10 significantly reduced KSHV viral genome amplification (**Fig 6H**) as well as expression of KSHV ORF50/Rta, K8/K-bZIP and ORF26 genes, which are immediate early, early and late lytic genes respectively, in TPA/NaB-treated HEK293.r219 cells (**Fig 6I**). Collectively, these results suggest that HOXA10 is a potent antiviral factor against KSHV lytic replication in contrast to its moderate effect on inhibiting EBV lytic replication.

### Identification of other isolated IRGs as antiviral factors against EBV and KSHV

Beyond IRG gene clusters subjected to EBV-mediated DNA hypermethylation and epigenetic silencing, we also examined the non-clusterous, isolated IRGs. We found that interleukin-1 receptor associated kinase 2 (IRAK2) and myelin and lymphocyte protein (MAL) are the two IRGs down regulated in EBVaGC patients (**S6A Fig**). DNA hypermethylation in these two loci were indicated by the higher beta values (**S6B, S6C, S6E and S6F Fig**). Pearson’s correlation analysis showed that there are significant inverse associations between DNA methylation and expression level of IRAK2 and MAL genes (**S6D and S6G Fig**).

**Fig 7:**
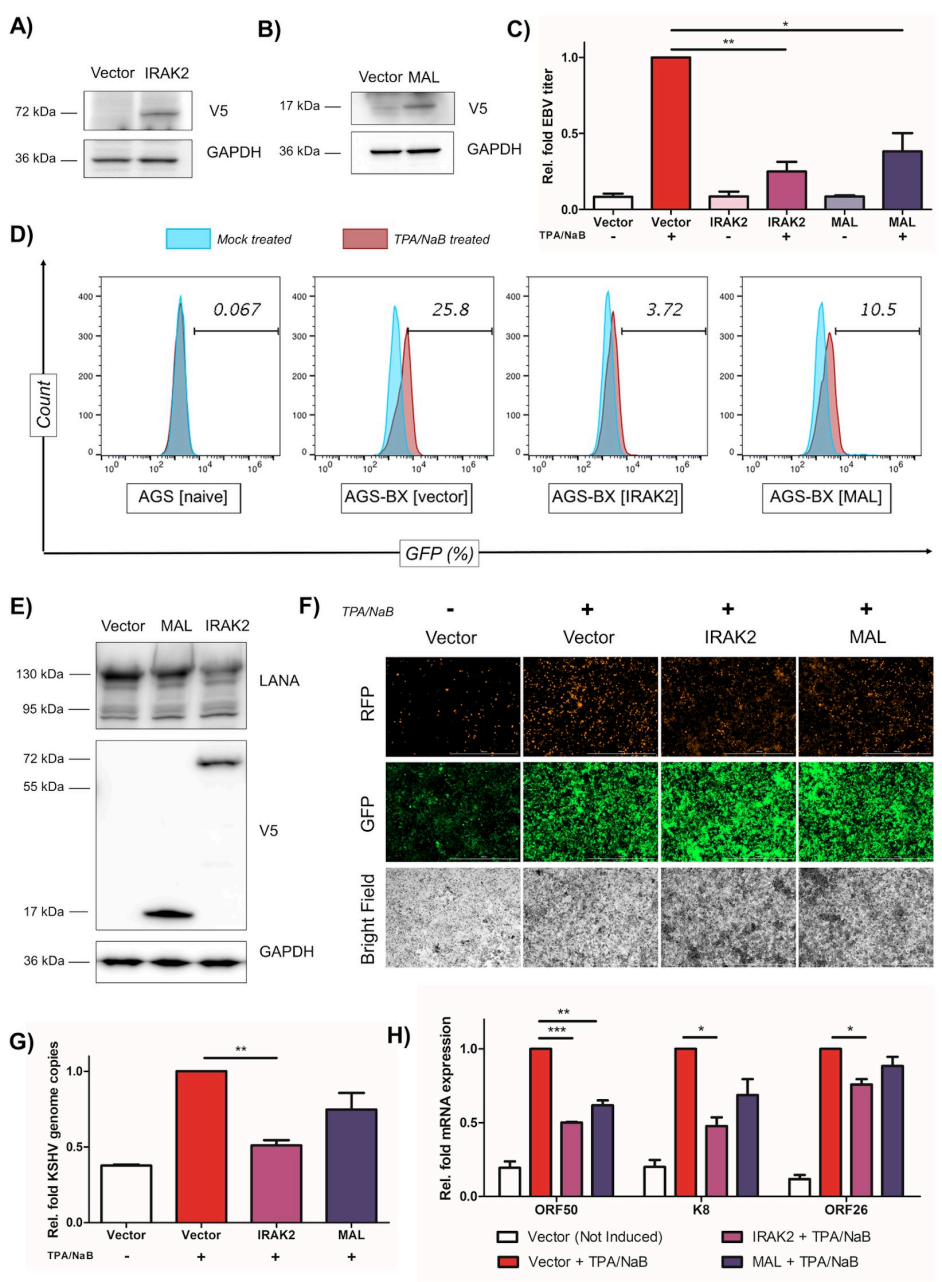
IRAK2 and MAL are two IRGs suppressing lytic replication of EBV and KSHV. (A, B) Protein expression of V5-tagged IRAK2 (A) or MAL (B) in stably transduced AGS-BX cells was verified by immunoblotting. (C) Newly produced EBV viruses from TPA/NaB treated or untreated AGS-BX cells stably expressing V5-IRAK2, V5-MAL, or the vector control, were titrated using Raji cells via flow cytometry. (D) Representative diagram of flow cytometry for (C). AGS cells were used to determine the background. (E) Protein expression of V5-tagged IRAK2 or MAL in transiently transfected HEK293.r219 cells was verified by immunoblotting. Protein expression of KSHV LANA was also probed. (F) Expression of GFP and RFP proteins in TPA/NaB treated or untreated HEK293.r219 cells transiently transfected with V5-IRAK2, V5-MAL, or the vector control, was visualized by fluorescence microscopy (scale bar = 1mm). (G) KSHV genome copies (ORF73/LANA DNAs) in TPA/NaB treated HEK293.r219 cells transiently transfected with V5-IRAK2, V5-MAL, or the vector control, were measured by qPCR assays at 24 hpi. (H) Expression of KSHV lytic genes (ORF50/Rta, K8/K-bZIP, and ORF26) in TPA/NaB treated or untreated HEK293.r219 cells transiently transfected with V5-IRAK2, V5-MAL, or the vector control, were measured by RT-qPCR assays at 24hpi. All of above results are based on n = 3 independent repeats and presented as mean ± SEM (* p<0.05; ** p<0.01; *** p<0.001, two-tailed paired Student t-test).

IRAK2 belongs to the IRAK family that also includes IRAK1, IRAK-M, and IRAK4, which is critical for the toll-like receptor (TLR) signaling and innate immunity [63–66]. MAL plays a role in T-cell development [67], and has been recently identified as a potential cancer biomarker, which undergoes severe promoter methylation and down-regulated expression in a wide range of cancers, such as colon cancer [68], cervical cancer [69,70], gastric cancer [71,72], head and neck cancer [73,74], and oral squamous cell carcinoma [75]. We further determined their antiviral potential against EBV, which has never been investigated previously. We stably transduced V5-tagged IRAK2 and MAL proteins in pLEX or the empty vector in AGS-BX cells, and the protein expression of IRAK2 and MAL were confirmed by immunoblotting (**Fig 7A and 7B**). These cells were treated with TPA/NaB to induce lytic replication and newly produced EBV virions were assessed using Raji cell infection assay. We observed a drastic reduction of EBV viral titer by ~75% and 60% respectively (**Fig 7C**) in cells that overexpressed IRAK2 or MAL., through the measurement of GFP-positive Raji cells (**Fig 7D**). Our further analysis showed that overexpression of IRAK2 and MAL does not affect EBV viral genome amplification or lytic gene expression (**S7 Fig**). These results suggested that IRAK2 and MAL interfere with later steps of EBV lytic replication instead, such as the assembly and/or budding of viral particle, leading to the overall reduction of EBV titer.

We also determined the antiviral potential of IRAK2 and MAL against KSHV. We transiently transfected IRAK2 and MAL in pLEX or the empty vector in HEK293.r219 cells, and their protein expression was verified by immunoblotting (**Fig 7E**). Overexpression of IRAK2 robustly reduced the RFP-expressing cells with GFP-expressing cells remained the same when compared to vector-transduced cells, while MAL had a weaker effect (**Fig 7F**). Consistently, overexpression of IRAK2 significantly reduced KSHV viral genome amplification (**Fig 7G**) as well as lytic gene expression, particularly ORF50 and K8 (**Fig 7H**) while MAL only weakly did so. Nevertheless, we demonstrate that non-clusterous, isolated IRGs subjected to EBV-mediated epigenetic dysregulation, such as IRAK2 and MAL, also possess the antiviral activity against lytic replication of both EBV and KSHV.

## Discussion

EBV has been implicated in a number of cancers with lymphoid and epithelial origins. Several studies further show that EBV infections drive the alterations of the host genome, leading to carcinogenesis [19,21,22,50,76]. EBV infection of epithelial and B cells modulate DNMT levels, which are influenced by the cell/tissue types and latency programs of EBV. There are three types of latency (I-III) dictated by distinct EBV gene expression patterns in various cancers. Several EBV-encoded viral proteins play a role in regulating the expression of DNMTs. LMP2A up-regulates the expression of DNMT1 and DNMT3B in EBV-associated gastric carcinoma (latency I) [21,77]. LMP1 up-regulates DNMT1, DNMT3A, and DNMT3B in EBV-associated nasopharyngeal carcinoma (latency II) [78,79]. In the Hodgkin’s lymphoma cell lines (latency II) as well as EBV-infected lymphoblastoid cell lines (latency III), it is more complicated that EBV increases the expression of DNMT3A through an uncharacterized viral gene(s) but down-regulates the expression of DNMT1 and DNMT3B expression through LMP1 [80]. It was recently shown that TET2 (Tet methylcytosine dioxygenase 2), a DNA demethylase, can interact with EBV EBNA2 protein and co-localizes with EBNA2-EBF1-RBP-jκ binding sites in cells [81]. In epithelial-origin cancers, EBV infection has been strongly associated with the gain of CpG methylation, and methylation of tumor suppressor genes such as p14, p16, IHH, TRADB, PTEN or SSTR1 is likely the key mechanism of oncogenesis [19,21,22,76].

**Fig 8:**
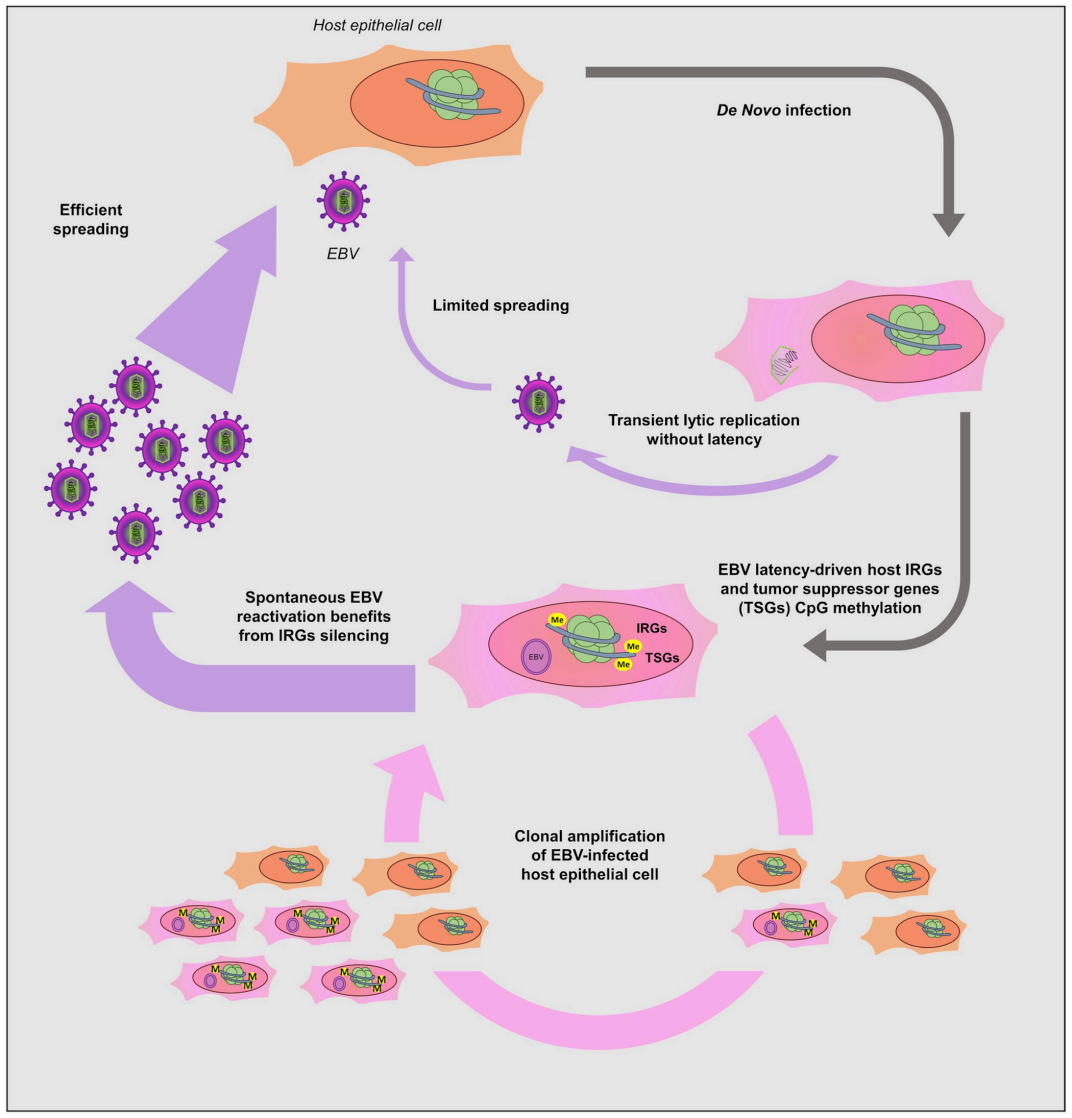
A working model describing the epigenetic silencing of antiviral IRGs and tumor suppressor genes by EBV through DNA hypermethylation. Once EBV infects the targeted host cells, i.e. gastric epithelium, host DNA methylation machineries are activated, leading to DNA hypermethylation across host genome. Certain gene groups are preferentially subjected to such EBV-mediated epigenetic dysregulation, including tumor suppressors and our newly characterized IRGs. Silencing of tumor suppressor genes (TSGs) would of course promote tumor development, while silencing of IRGs, particularly those possessing antiviral activities, demonstrated by our studies, would benefit EBV viral propagation and spreading. Besides, silencing of IRGs may also benefit EBV oncogenesis, since some IRGs are reported to exert antitumor potential using the same immune signaling as its antiviral function. Therefore, massive DNA methylation of IRG loci would bring dual benefits to EBV, an oncovirus, not only favoring EBV lytic replication but also promoting malignant transformation of infected cells. The antiviral activities of some IRGs may not just limit to EBV, but extend to KSHV, another gamma-herpesvirus.

Through analyzing the transcriptome and DNA methylome analysis of EBVaGC, we identified a unique feature that IRGs are another group of genes, besides tumor suppressors, which are preferentially silenced by EBV through DNA hypermethylation (**Figs 1 and 2**). Given the antiviral nature of IRGs, it is plausible that silencing of these genes would favor EBV lytic replication and viral spreading. This hypothesis is further proved by our results demonstrating the previously unreported anti-EBV activity of selected IRGs (**Figs 5–7**). In recent years, the significance of EBV lytic replication in carcinogenesis has been described in several studies. Expression of EBV earliest lytic gene, BALF3, is linked to host genome instability by inducing micronuclei and DNA strand breaks in nasopharyngeal carcinoma (NPC) [82]. In another study by Fang et al, both oncogenes expression and genome instability increase following recurrent lytic reactivation of EBV in NPC [83]. These studies shed light on the role of viral lytic replication in EBV pathogenesis beyond viral propagation. EBV infection of epithelial cells, such as NPC and EBVaGC, is lytic in nature. In these cases, we speculate that EBV-induced epigenetic silencing of IRGs would maintain the lytic replication of EBV at a certain level and directly promoting tumorigenesis beyond the production of progeny viruses for spreading (**Fig 8**). Such dysregulation of IRGs by EBV is probably a virus-driven event, since we observed similar pattern of IRGs in both EBV-infected GC and BL cells (**Fig 4**). We also noticed that the IRGs we studied (MT1H, HOXA10, IRAK2, MAL) also suppress KSHV lytic replication (**Figs 6 and 7**). KSHV is another member of *gammaherpesviridae* family besides EBV. Although it remains to be confirmed, it is likely that these IRGs are also subjected to DNA hypermethylation since it has been shown that KSHV also hijacks host DNA methylation regulators [18]. KSHV may use the same strategy to dysregulate IRGs, favoring viral propagation and oncogenesis.

EBV-mediated silencing of IRGs is an effective viral strategy to generate dual benefits in viral oncogenesis, since some IRGs, including those we studied, possess both antiviral and antitumor activities. Metallothioneins play a critical role in maintenance of zinc homeostasis and act as scavenger for reactive oxygen species, which explains their role in innate immunity [84] and carcinogenesis [41]. Down-regulation of MT1 proteins have been observed in esophageal squamous cell carcinoma [85,86], hepatocellular carcinoma [39,87], papillary thyroid carcinoma [88], and prostate cancer [89], suggesting their role as tumor suppressor. It is known that oxidative stress (OS) signals are induced by herpesvirus infection, which favors viral replication [90]. Recent evidence showed that level of reactive oxygen species (ROS) increases during EBV infection [91]. MT1 proteins are shown to protect the cells against OS through its antioxidant properties [92], which may explain its anti-EBV activities. Given that OS also promotes tumorigenesis likely through induction of DNA oxidative damage, it is highly possible that the antiviral and antitumor immunity of MT1 utilize the same mechanism. Our results indicated that MT1 gene cluster is hypermethylated in EBVaGC (**Fig 3A**), and that indeed there are redundant anti-EBV functions among several MT1 genes (**Fig 5**). This is likely an effective method for EBV to target the whole MT1 gene cluster to abolish the antiviral and antitumor activities of MT1 genes.

In our study, we confirmed the antiviral activities of HOXA10 against both EBV and KSHV (**Fig 6**), reminiscent of the recent finding that HOXA10 suppresses HBV as well [59]. This study also identified that HOXA10’s suppressive effect is due to the inhibition of the p38 MAPK/STAT3 signaling pathway through SHP-1-catalyzed dephosphorylation. Indeed, induction of the p38 MAPK signaling promotes the EBV lytic replication [60]. Lytic replication of KSHV also requires the p38 MAPK signaling [62,93]. Moreover, ROS-induced KSHV reactivation depends on p38 MAPK as well [94], indicating that the MT1G and HOXA10 mediated antiviral activities may cross talk with each other through inhibition of OS and p38 signals that are connected. HOXA10 is reported as either an oncogene or a tumor suppressor, depending on the cancer types. In testicular and prostate cancers, HOXA10 plays a tumor suppressive role [95,96]. In gastric cancer, HOXA10 is one of the most significant DNA methylation markers [97,98], but contradictorily it seems required for cell proliferation [99,100]. The direct contribution of HOXA10 to gastric cancer needs to be further clarified. In addition, chronic infection of the stomach mucosa with cytotoxin-associated gene (CagA) -positive strain of *Helicobacter pylori* is highly associated with gastric cancer [101–103]. Recent studies have revealed the symbiotic relationship between *H*. *pylori* and EBV in gastric carcinoma [104,105]. SHP-1 was found to dephosphorylate CagA and thus mitigate its oncogenic potential while EBV infection leads to the SHP-1 down-regulation via promoter DNA methylation, which augments the oncogenic activities of *H*. *pylori* [105]. It will be compelling to investigate whether HOXA10 is involved.

Promoter methylation and transcriptional silencing of MAL protein has been reported for numerous cancers, such as colon cancer [68], cervical cancer [69,70], gastric cancer [71,72], head and neck cancer [73,74], and oral squamous cell carcinoma [75]. Cervical cancer is another virus-driven cancer, and human papillomavirus (HPV) type 16 and 18 are responsible for 70% of those cases. Like EBVaGC, HPV infection correlates with the silencing of MAL in cervical cancer [69,70]. It will be intriguing to test whether MAL also suppresses HPV replication similar to its antiviral activity against EBV and KSHV (**Fig 7**), which may present a general theme in viral oncogenesis. We also identified that IRAK2 is subjected to EBV-induced epigenetic down-regulation in EBVaGC, and that its expression suppresses lytic replication of EBV and KSHV (**Fig 7**). IRAK2 has emerged as a prominent member of the TLR response [63,65]. Hou et al [64] recently described the role of IRAK2 in RIG-I signaling and type I interferon production. EBV-encoded small RNAs (EBERs) are ubiquitously expressed in EBV-infected cells, which is recognized by RIG-I that leads to the activation Type I interferon [106]. Type I interferon can act in a paracrine manner to further activate the JAK/STAT pathway in the neighboring cells, which results in the induction of interferon stimulated genes to protect these cells from EBV infection. Thus, it is plausible that epigenetic silencing of IRAK2 by EBV would result in the pro-viral benefits by promoting EBV viral replication and increasing the susceptibility of nearby cells to EBV infection within the microenvironment. Recently, IRAK2 has also been described as a potential tumor suppressor in colon cancer that counterbalances oncogenic Smurf1 by phosphorylation of its threonine residues and promoting its self-degradation by ubiquitylation [107].

Contrary to the fact that the majority of IRGs (381/497, 76.7%) is down-regulated in EBVaGC, we also observed that a subset of IRGs (116/497, 23.3%) is up-regulated (**Fig 1A**). Interestingly, for the majority of up-regulated IRGs (89/116, 76.7%), their loci are DNA hypermethylated in EBVaGC (**Fig 2B**). DNA methylation occurring to gene body rather than promoter region may explain the upregulation of these IRGs [33–35]. Indeed, our analysis unraveled that DNA methylation level at gene body *vs* promoter region of up-regulated, DNA hypermethylated IRGs is higher than that of down-regulated, DNA hypermethylated IRGs (**Fig 2C**). Other epigenetic mechanisms, such as histone modifications, may also profoundly interfere with DNA methylation and overcome its silencing effect [108]. In this study, Fürst et al. showed that increased CpG methylation of a single site alters the binding of transcriptional repressor TGIF1 to the host DNA and lowers the local histone H3 occupancy, which results in the increase of ESR1 mRNA expression. In addition, there is a growing body of evidence that DNA hypermethylation links to positive gene expression as demonstrated by a recent systemic study in prostate cancer [109], supported by other studies showing that certain transcription factors preferentially bind to methylated DNA [110,111]. Beyond the transcriptional level, EBV infection has the potential to trigger multiple innate immune signaling pathways. The gene expression pattern reflects the combinatory consequence of epigenetic silencing and innate immune activation. For instance, EBV infection triggers type 1 interferon response via RIG-I [106] and TLR signaling pathway [112]. Therefore, certain DNA hypermethylated IRGs might be up-regulated due to immune response triggered by the on-going EBV infection. EBV also develops several other mechanisms to directly interfere with immune response via BART miRNA [113], as well as latent and lytic proteins, such as LMP2 [114] and Zta [115]. Thus, CpG methylation is just one viral mechanism used to dampen the immune response triggered by EBV. Overall, these observations highlight the complexity of host-EBV interactions at multiple levels that delicately regulate the expression of IRGs. For those up-regulated IRGs, it remains to be determined whether they have anti-viral potential. It has been shown that activation of certain IRGs, such as caspase-3, -6, and -8, actually benefits EBV infection [116]. On the other hand, induction of IRGs may be a part of inflammatory response, which favors viral tumorigenesis in EBVaGC [29]. Thus, the tumor-promoting benefits due to the upregulation of certain IRGs may surpass their adverse effect on viral replication if there is any. Nevertheless, our analysis indicated that the majority of IRGs is down-regulated due to EBV-induced global epigenetic silencing of host transcriptomic landscape, which may constitute an overall antiviral cellular environment, despite the upregulation of a small subset of IRGs.

To summarize, our study recognized that EBV preferentially methylates the loci of IRGs and epigenetically silence their expression in EBVaGC. We further demonstrated that certain examples of IRGs in gene cluster (MT1G/MT1H, HOXA10) or those isolated ones (MAL, IRAK2) possess antiviral activities against EBV and KSHV. These results supported that EBV-induced epigenetic silencing of IRGs would benefit viral lytic replication, which promotes viral propagation and spreading as well as viral oncogenesis. Furthermore, given that innate immunity plays an equally important role in controlling viral infection and tumor development, we expect that systematic suppression of IRGs by oncoviruses, such as EBV, would also favor the proliferation and malignant transformation of infected cells. Future similar studies of other oncoviruses, such as KSHV and HPV, will validate whether it is a general strategy utilized by oncoviruses to promote tumors. Additionally, our preliminary result of DNMT inhibitors (**Fig 4E and 4F**) indicated that these epigenetic-modulating reagents could be useful for reversing EBV-induced DNA hypermethylation of host genes, thus restoring the expression of antiviral/antitumor IRGs, and resetting the cellular environment towards better controlling viral replication and oncogenesis.

## Materials and methods

### Bioinformatics analysis

RNAseq data was obtained from TCGA data portal (https://portal.gdc.cancer.gov). EBV-positive and EBV-negative samples were selected to contain tumors with similar composition of TNM (Tumor, Node, Metastasis) stages. EBV-positive samples were further confirmed by detection of EBER and EBNA1 transcripts. Differential expression analysis was performed by using R implementation of the DESeq2 package [117], which uses negative binomial distribution to model read counts. Each count is normalized by sample-specific size factors, which is the median ratio of gene count to geometric mean per gene. Differentially expressed genes are sorted based on the adjusted p-value (FDR correction) with cutoff of 0.05. Methylation data was obtained from TCGA data portal. The data were collected from the Infinium II HumanMethylation 450 array, which uses red and green signals to screen more than 450,000 CpG probes across human genome (Illumina, San Diego, CA). Differential methylation analysis was performed by using Minfi package (implemented in R) [118]. It converts red and green channels into a matrix of methylated and unmethylated signals and performs subset-quantile within array normalization (SWAN) for eventual differential methylation analysis by limma (linear models for microarray) package [119]. Differentially methylated region was annotated by integrating Minfi and DMRcate packages [120] in R. Functional enrichment analysis was performed by using DAVID (https://david.ncifcrf.gov) with adjusted p-value threshold (FDR method) of 0.05. Circos software was used to visualize gene expression and DNA methylation pattern [121].

### Detection of EBV lytic transcripts

To determine EBV lytic gene expression in EBVaGC samples, unmapped reads of RNAseq data were extracted and aligned to EBV reference genome (Akata strain, https://github.com/flemingtonlab/public/tree/master/annotation). Transcripts of EBV lytic genes were counted from those aligned reads by using expectation maximization algorithm (RSEM) to obtain the TPM (transcript per million) value. For selected EBV lytic genes, percentage of EBVaGC samples expressing these viral genes was illustrated.

### Cells

AGS and AGS/BX cells were maintained in F-12 media complemented with 10% FBS. HEK293T and HEK293.r219 cells were maintained in DMEM supplemented with 10% FBS. Raji, Akata, and Akata/BX cells were maintained in RPMI supplemented with 10% FBS. EBV-negative Akata cell line was clonally selected for the loss of viral episome from the original EBV-positive Akata Burkitt’s Lymphoma cell line [48], which was subsequently re-infected with EBV BX strain and selected with neomycin. Recombinant EBV-BX was maintained using G418 (Invitrogen) selection at 500μg/mL and recombinant KSHV.219 was maintained using Puromycin (Invitrogen) selection at 5μg/mL. HEK293T.EBV.BacGFP cell line was maintained as previously described [122].

### Compounds and antibodies

DNA methylation inhibitors, 5-Azacytidine and 5-Aza-2’-deoxycytidine, were purchased from Sigma Aldrich (Cat# A1287 and A3656, respectively), and dissolved in acetic acid according to the manufacturer’s recommendations. Zn^2+^ solution was prepared in H_2_O from heptahydrate zinc sulfate (Sigma Aldrich, Cat# Z0251). The antibodies used in this study were anti-V5-HRP (Invitrogen, Cat# R961-25), anti-GAPDH (SantaCruz, Cat# 32233), anti-Mouse-HRP (Invitrogen, Cat# 31430), anti-Rat-HRP (Invitrogen, Cat#A18739) and anti-KSHV_LANA (Advanced Biotechnologies, cat# 13-210-100) antibodies.

### Plasmids

ORFs of MT1H (NM_005951.2), MT1M (NM_176870.2), MT1X (NM_005952.3), IRAK2 (NM_001570.3), MAL (NM_002371.2) in pLEX307 vector were picked from the MISSION TRC3 Human LentiORF Collection (Sigma) (Clones ID: TRCN0000474764, TRCN0000470527, TRCN0000475214, TRCN0000472831, and TRCN0000473853, respectively). ORF of MT1F (NM_005949.3) in pDONR223 vector was picked from the same library (Clone ID: ccsbBroadEn_01041) and cloned in pLEX307 vector (Addgene#41392) using the LR clonase (Invitrogen). ORFs of MT1G and HOXA10 were purchased from Genscript (Clone ID: OHu59080D, OHu27157, respectively). These ORFs were PCR amplified using the following primers: MT1G, forward 5’ GGG GAC AAC TTT GTA CAA AAA AGT TGG CAT GGA CCC CAA CTG CTC CT 3’, reverse 5’ GGG GAC AAC TTT GTA CAA GAA AGT TGG GCA GGC GCAG CAG CTG CAC 3’; HOXA10, forward 5’ GGG GAC AAC TTT GTA CAA AAA AGT TGG CAT GTC AGC CAG AAA GGG CTA TC 3’, reverse 5’ GGG GAC AAC TTT GTA CAA GAA AGT TGG GCA GGA AAA ATT AAA GTT GGC TGT 3’. The PCR amplicons were initially cloned into pDONR221 vector using BP clonase (Invitrogen), and then into the pLEX307 vector (Addgene#41392) using LR clonase. All plasmids were verified by sanger sequencing. Control pLEX307 vector was generated by inserting a short oligonucleotide coding for FLAG peptide into the pLEX307 vector (empty vector). HEK293T cells were transfected with lentiviral packaging vectors, psPAX2 (Addgene#12260) and pMD2G (Addgene#12259), by using Turbofect (Invitrogen) transfection reagent according to the manufacturer’s instructions. Media was replaced within 12hrs post-transfection, harvested at 48 and 72hrs post-transfection, and filtered through 0.45μm membrane filters (Millipore). Transduction of AGS-BX cells was performed in presence of Polybrene (8ug/mL) for 24hrs (MOI = 5), and puromycin (2μg/mL) was added at least 2 days post-transduction for selection of stable cell lines.

### Titration of EBV viruses

EBV titration assay using Raji cells was adapted from the previously published protocols [123,124] with slight modifications. Briefly, AGS-BX stable cell lines (2x10^5^ cells) were induced with TPA and NaB (20ng/mL and 1.5mM, respectively) for 24hrs. Cells were washed, and kept in culture with fresh culture media for an additional 48hrs. Media was collected, filtered through 0.45um syringe filters, and added to Raji cells (4x10^5^ cells) cells at a ratio of 1:1 and 1:3 (supernatant *vs* fresh RPMI) for EBV *de novo* infection. At 24hrs post-infection, Raji cells were treated with TPA and NaB (20ng/mL and 3mM, respectively), and kept in culture for an additional 24hrs, followed by the flow cytometry assay using an Accuri C6 Plus (BD Biosciences). Percentage of GFP-positive Raji cells was quantified and normalized to that in the condition of AGS-BX cells stably expressing empty vector. Forward Scatter (FS) and Side Scatter (SS) were used for gating of single cells. Analysis was performed using the FlowJo v10 software.

### Real-time PCR

Total RNA was extracted using the NucleoSpin RNA extraction kit (Macherey-Nagel) according to the manufacturer’s instructions. cDNA was prepared using iScript (BioRad) and subjected to the real-time PCR analysis on a CFX96 instrument (BioRad), by using (2x) SYBR Green Supermix (BioRad). Data was analyzed by using the ΔΔCt method with GAPDH as an internal control. Primers used in this study are summarized in **S3 Table**. For analyzing EBV genome copies, genomic DNA was extracted using Qiagen DNA Easy extraction kit. Relative EBV copy number was measured by real-time PCR analysis by using EBNA1 and GAPDH primers (**S3 Table**).

### Fluorescence imaging

Fluorescence was acquired on a BioTeK plate reader by using its GFP and RFP channels. Expression of GFP and RFP proteins are respectively driven by the eIF1a promoter and KSHV lytic PAN promoter [58].

### Cell viability

Cell viability was assessed by using CellTiter-Glo (Promega, Cat# G7570) according to the manufacturer’s instructions.

### siRNA knockdown

HEK293.r219 cells was reversely transfected with siRNAs using Lipofectamine RNAimax (Life Technologies) following manufacturer’s instructions. Silencer select MT1H siRNAs (s9014 and s9015 labeled as si1 and si2 respectively, Life Technologies) or control siRNA (CNT) were used at a final concentration of 10 nM. KSHV lytic reactivation was induced by transfection of KSHV ORF50 using FuGene6 (Promega) according to manufacturer’s instructions. Fluorescence images were acquired on a BioTeK plate reader using GFP, RFP, and DAPI channels. Cell nucleus were stained using Hoechsst dye (Life Technologies) and used for normalization. Expression of GFP and RFP proteins are driven by the eIF1a promoter and KSHV lytic PAN promoter respectively [58].

### Methylated DNA immunoprecipitation (MeDIP)

The MeDIP method was adapted from a previous report [125]. In brief, AGS and AGS-BX cell pellets were digested for overnight with proteinase K at 50°C in the proteinase K digestion buffer (100mM NaCl, 10mM TrisCl, 25 mM EDTA, 0.5% SDS, and 0.1mg/ml proteinase K, pH = 8). Genomic DNA was extracted using a phenol/chloroform/isoamyl alcohol (25:24:1) mixture, and sheared with a sonication device (50% amplitude, 2m total with 5s on and 2s off). Sheared DNA samples were denatured, pre-cleared with protein A/G magnetic beads (ThermoFisher, Cat#88802), and incubated with 1ug of anti-m5C antibody (Clone 33D3, cat#A-1014, Epigentek) or anti-mouse IgG (Cat# SC-2025, SantaCruz Biotech) per 4ug DNA in the MeDIP precipitation buffer (10mM NaH_2_PO_4_, 140mM NaCl, 0.05% Triton X-100, pH = 7) at 4°C for overnight. Mixture was further incubated with protein A/G magnetic beads for 1h at 4°C, followed by washing with MeDIP precipitation buffer for four times. Beads were collected and digested with proteinase K for 3h at 50°C. Precipitated DNA was extracted with phenol/chloroform/isoamyl alcohol (25:24:1). DNA pellets were resuspended in the TE buffer and subjected to qPCR analysis. An aliquot of pre-cleared sheared DNA was saved as input, and subjected to similar proteinase K digest and DNA extraction. Results were presented as the relative fold enrichment.

### Chromatin immunoprecipitation (ChIP)

ChIP was performed using the MagnaChIP HiSens kit (Cat# 17–10460, EMD Millipore) following the manufacturer’s recommendations. Anti-V5 (Cat# R960-25, Invitrogen) or anti-Mouse IgG (Cat# SC-2025, SantaCruz Biotech) antibody were used. Results were presented as the relative fold enrichment.

### Statistical analysis

Statistical analysis was performed in GraphPad PRISM 5, R and Excel. Data are presented as mean ± SEM of independent experiments (n = 3). * p<0.05; ** p<0.01; *** p<0.001, except specified otherwise.

## Supporting information

S1 Fig (A) Gene ontologies (biological processes) that are associated with up-regulated IRGs. (B) Bar plot showed the normalized mRNA expression for selected top IRGs up-regulated in EBVaGC vs non-EBV GC. (C) Bar graph summarizes the percentage of EBVaGC samples that express transcripts of selected EBV lytic genes (immediate early, early, late genes). Detection threshold is ≥1 TPM (transcript per million).
(TIF)

S2 Fig (A) Venn diagram showed the number of differentially methylated (DE) non-IRGs (grey circle) for both down-regulated non-IRGs (left, blue circle) and up-regulated non-IRGs (right, red circle). (B) Loci of methylation for dysregulated IRGs are shown in the stacked bar plot. Proportion of each methylation sites in the whole array is included. CpG Island was the predominant methylation site for the down-regulated IRGs.
(TIF)

S3 Fig (A) Viability of AGS-BX cells treated with 5-azacytine, 5-aza-2-deoxycytine, or vehicle control, was measured by Cell Titer Glo. The value of vehicle control was set up as 1. (B) CpG m5C methylation at HOXA10 and MT1G promoter regions in AGS and AGS-BX cells was measured by MeDIP-qPCR. Primers target the promoter region corresponding to the TCGA 450K CpG array probe loci with hypermethylation in EBVaGC. Representative results of 3 independent experiments are displayed as relative fold enrichment. Data are presented as mean ± SEM of 3 technical repeats (* p<0.05, two-tailed paired Student t-test).
(TIF)

S4 Fig pLEX307 vector expressing MT1G-V5 (A) or HOXA10-V5 (B), or vector control, was transiently transfected in HEK293T.EBV.BacGFP cells. At 48h post of transfection, cells were treated with TPA/NaB for an additional 48h to induce EBV lytic reactivation or untreated to maintain the basal level. Cell lysate was prepared and subjected to ChIP-qPCR. Primers target the promoter region of various EBV lytic genes. Representative results of 2 independent experiments are displayed as relative fold enrichment. Data are presented as mean ± SEM of 3 technical repeats (* p<0.05, one-tailed paired Student t-test).
(TIF)

S5 Fig (A) Expression of GFP and RFP proteins in ORF50-transfected HEK293.r219 cells treated with MT1H siRNAs (si1, si2) or control siRNA (CNT) was visualized by fluorescence microscopy (scale bar = 1mm). With GFP at the downstream of eF1α promoter and RFP gene downstream of KSHV lytic PAN promoter, expression of these fluorescent protein indicates KSHV latent infection and lytic reactivation respectively. (B) Percentage of RFP-positive cells undergoing KSHV lytic reactivation was calculated and normalized to the control siRNA (CNT) (n = 3). Results are presented as mean ± SEM (* p<0.05; ** p<0.01; *** p<0.001, two-tailed paired Student t-test). (C) Knockdown efficiency of MT1H siRNAs (si1, si2) in HEK293.r219 cells was analyzed by RT-qPCR at 3 days post of transfection. Results are based on n = 3 independent repeats and presented as mean ± SEM (* p<0.05, two-tailed paired Student t-test).
(TIF)

S6 Fig (A) Bar plot showed the normalized mRNA expression for IRAK2 and MAL in EBVaGC vs non-EBV GC. (B, E) Heat map showed the beta value of each CpG probe located within IRAK2 (B) and MAL (E). EBV-negative GC samples (n = 25), upper panel; EBVaGC samples (n = 23), lower panel. (C, F) Box plot showed the distribution of beta values for all CpG probes located within IRAK2 (C) and MAL (F) in EBVaGC and non-EBV GC samples. Results were based on n = 23 (EBV +) and 25 (EBV -) samples and presented as mean ± S.D. (* p<0.05; ** p<0.01; *** p<0.001, Wilcoxon signed-rank test). (D, G) Correlation plot showed the inverse relationship of mRNA expression and methylation beta values for IRAK2 (D) and MAL (G). P-value and Pearson’s correlation coefficient are indicated.
(TIF)

S7 Fig (A) EBV genome copies (EBNA1 DNAs) in TPA/NaB treated AGS-BX cells stably expressing V5-IRAK2, V5-MAL, or the vector control, were measured by qPCR assays at 24 hpi. The relative level of EBNA1 DNAs in TPA/NaB treated AGS-BX cells transduced with the vector control was set up as 1. (B) Expression of EBV lytic genes (BZLF1, BRLF1, BMRF1, BALF5, BNRF1, BcLF1, BLLF1) in TPA/NaB treated or untreated AGS-BX cells stably expressing V5-IRAK2, V5-MAL, or the vector control, were measured by RT-qPCR assays at 24hpi. Results are based on n = 3 independent repeats and presented as mean ± SEM (* p<0.05, two-tailed paired Student t-test).
(TIF)

S1 Table Complete curated list of published IRGs (4215 genes) we manually compiled and used throughout the study (including appropriate references). (XLSX)

S2 Table List of the differentially expressed (DE) all genes and IRGs (EBVaGC vs EBV-negative GC), as well as the differentially methylated (DM) IRGs. (XLSX)

S3 Table List of primers used in this study. (XLSX)
