# Supplementary figures and images for: Profiling of immune related genes silenced in EBV-positive gastric carcinoma identified novel restriction factors of human gammaherpesviruses
Source: PLoS Pathog. 2020 Aug 25;16(8):e1008778. doi: 10.1371/journal.ppat.1008778 (PMC7473590; doi:10.1371/journal.ppat.1008778)

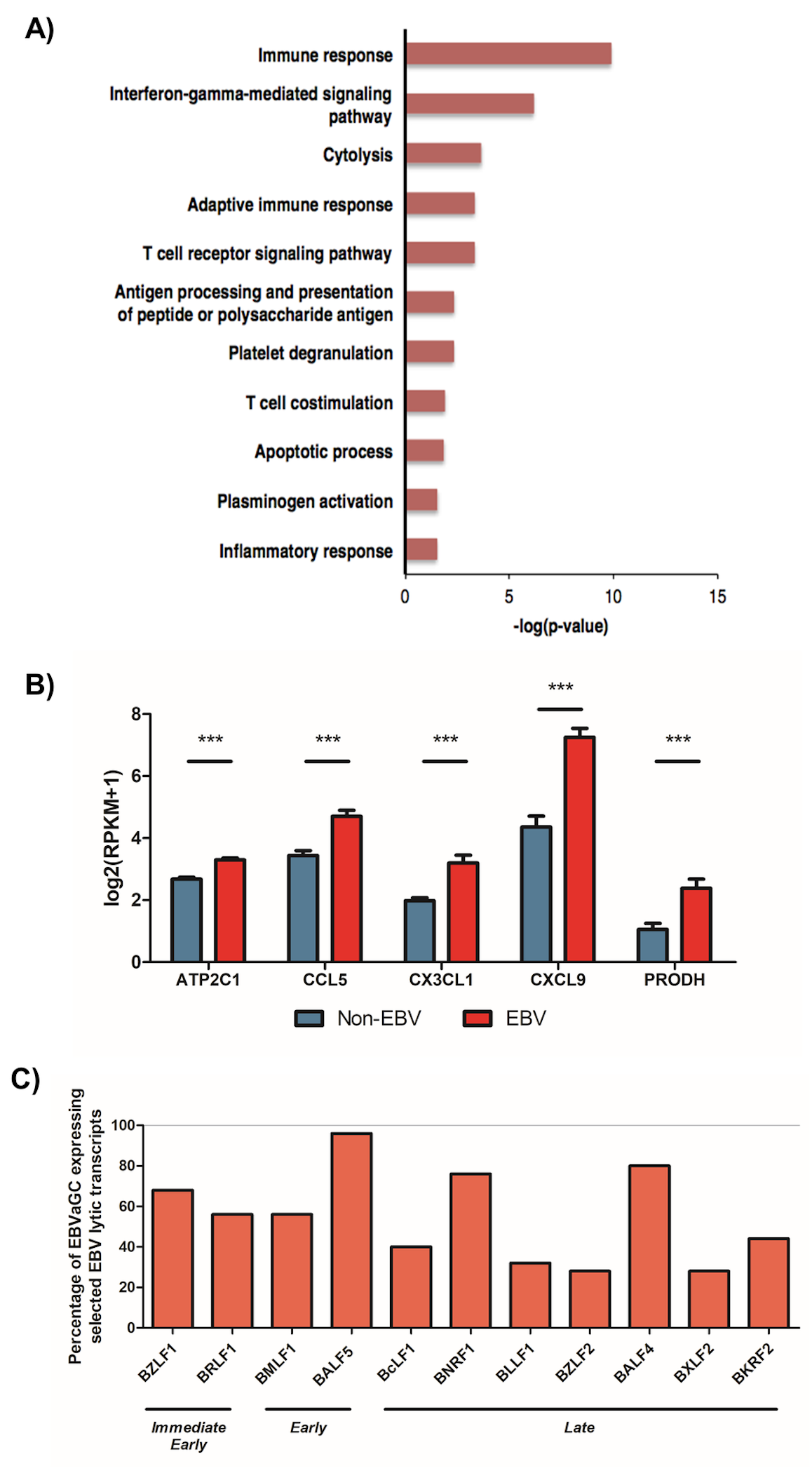

Supplement: S1 Fig — (A) Gene ontologies (biological processes) that are associated with up-regulated IRGs. (B) Bar plot showed the normalized mRNA expression for selected top IRGs up-regulated in EBVaGC vs non-EBV GC. (C) Bar graph summarizes the percentage of EBVaGC samples that express transcripts of selected EBV lytic genes (immediate early, early, late genes). Detection threshold is ≥1 TPM (transcript per million). (TIF) [file ppat.1008778.s001.tif]

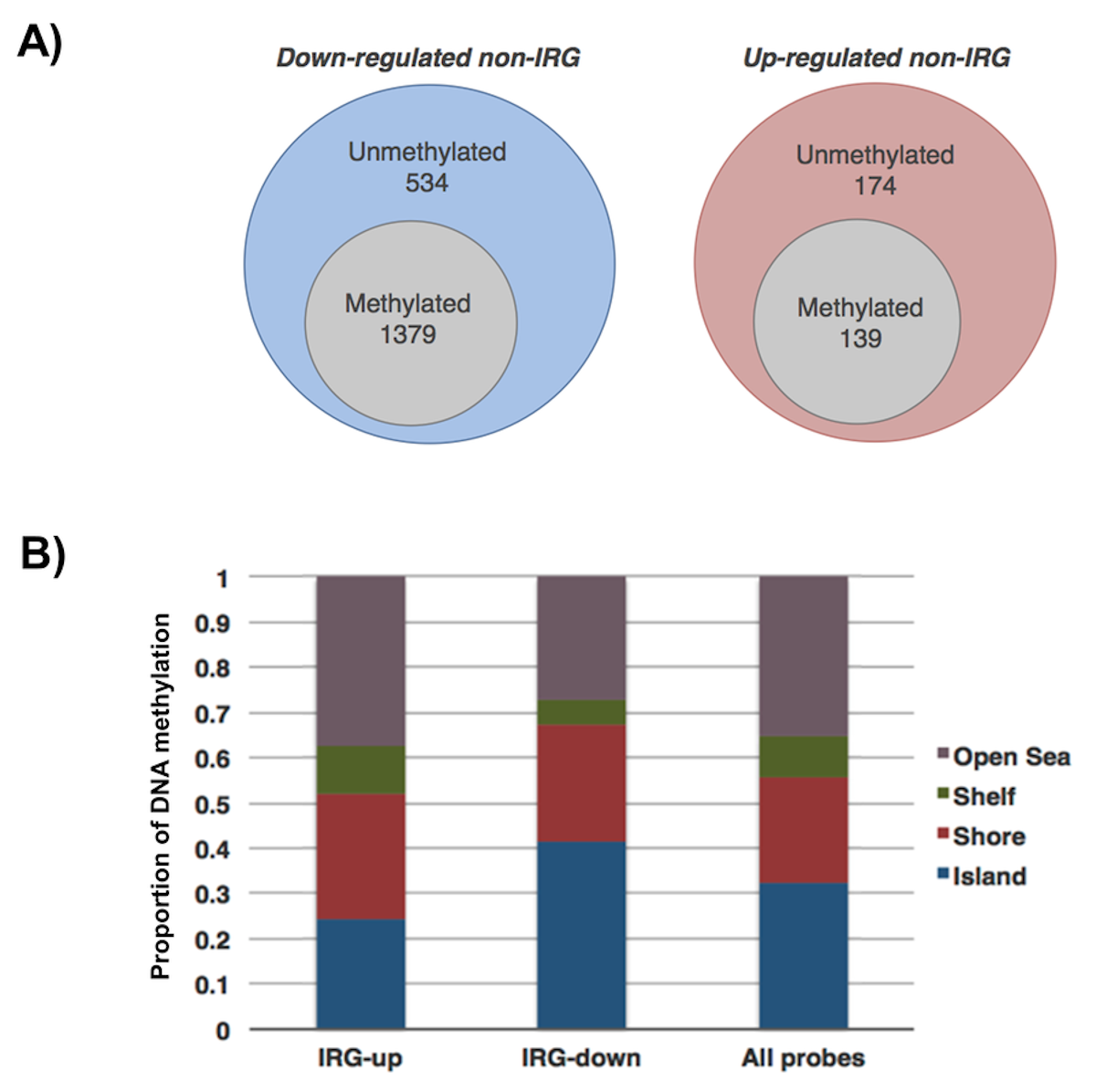

Supplement: S2 Fig — (A) Venn diagram showed the number of differentially methylated (DE) non-IRGs (grey circle) for both down-regulated non-IRGs (left, blue circle) and up-regulated non-IRGs (right, red circle). (B) Loci of methylation for dysregulated IRGs are shown in the stacked bar plot. Proportion of each methylation sites in the whole array is included. CpG Island was the predominant methylation site for the down-regulated IRGs. (TIF) [file ppat.1008778.s002.tif]

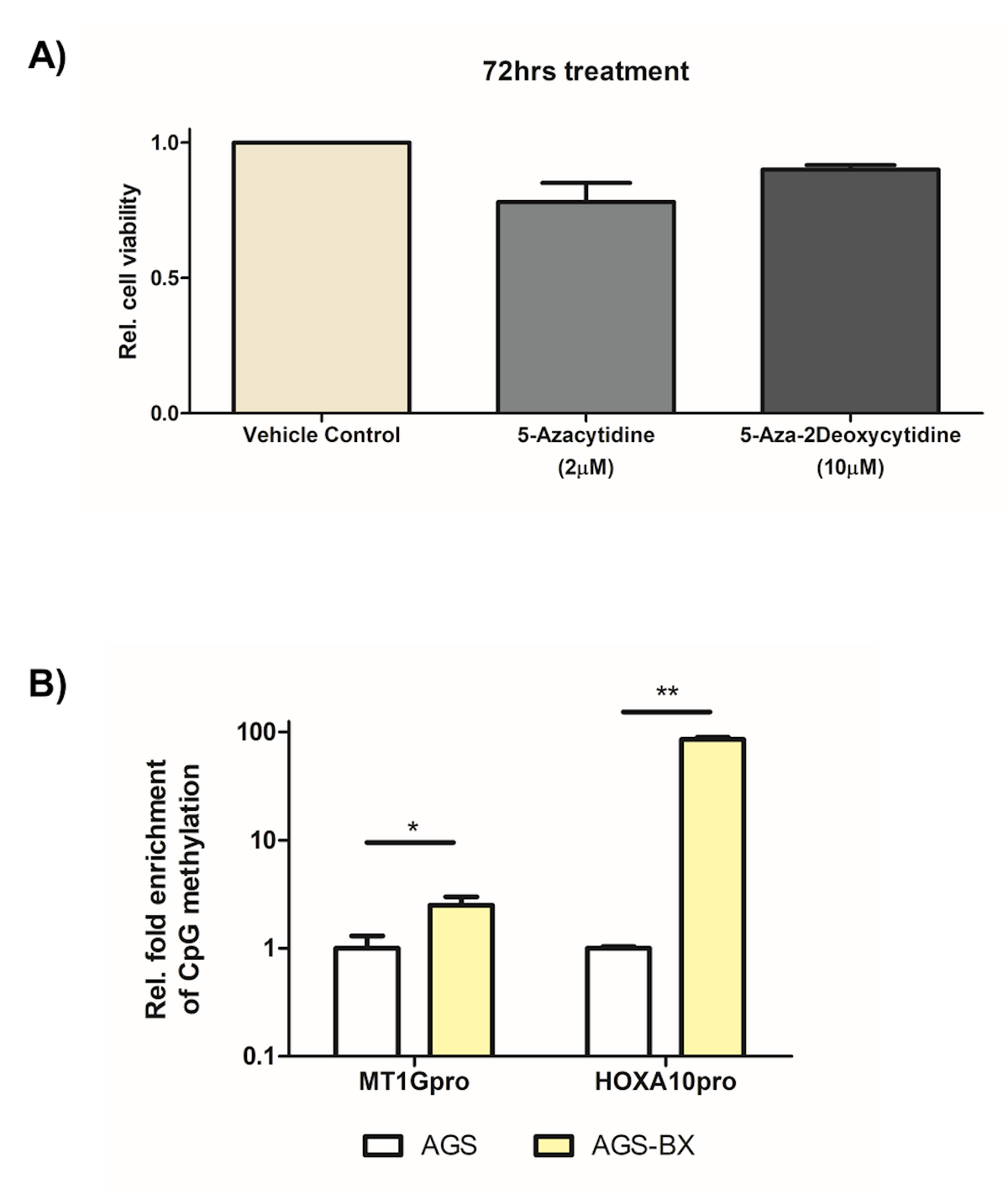

Supplement: S3 Fig — (A) Viability of AGS-BX cells treated with 5-azacytine, 5-aza-2-deoxycytine, or vehicle control, was measured by Cell Titer Glo. The value of vehicle control was set up as 1. (B) CpG m5C methylation at HOXA10 and MT1G promoter regions in AGS and AGS-BX cells was measured by MeDIP-qPCR. Primers target the promoter region corresponding to the TCGA 450K CpG array probe loci with hypermethylation in EBVaGC. Representative results of 3 independent experiments are displayed as relative fold enrichment. Data are presented as mean ± SEM of 3 technical repeats (* p<0.05, two-tailed paired Student t-test). (TIF) [file ppat.1008778.s003.tif]

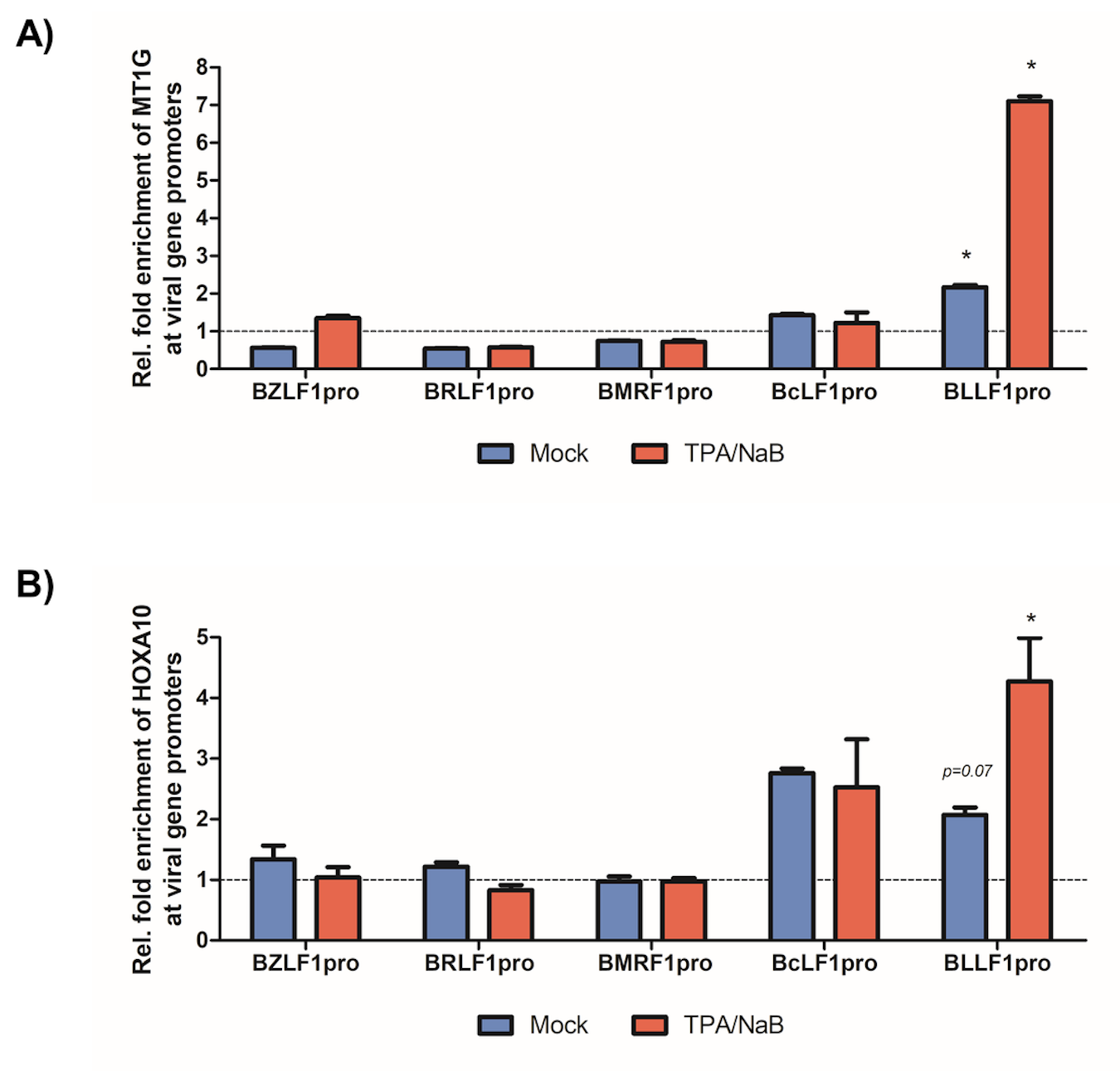

Supplement: S4 Fig — pLEX307 vector expressing MT1G-V5 (A) or HOXA10-V5 (B), or vector control, was transiently transfected in HEK293T.EBV.BacGFP cells. At 48h post of transfection, cells were treated with TPA/NaB for an additional 48h to induce EBV lytic reactivation or untreated to maintain the basal level. Cell lysate was prepared and subjected to ChIP-qPCR. Primers target the promoter region of various EBV lytic genes. Representative results of 2 independent experiments are displayed as relative fold enrichment. Data are presented as mean ± SEM of 3 technical repeats (* p<0.05, one-tailed paired Student t-test). (TIF) [file ppat.1008778.s004.tif]

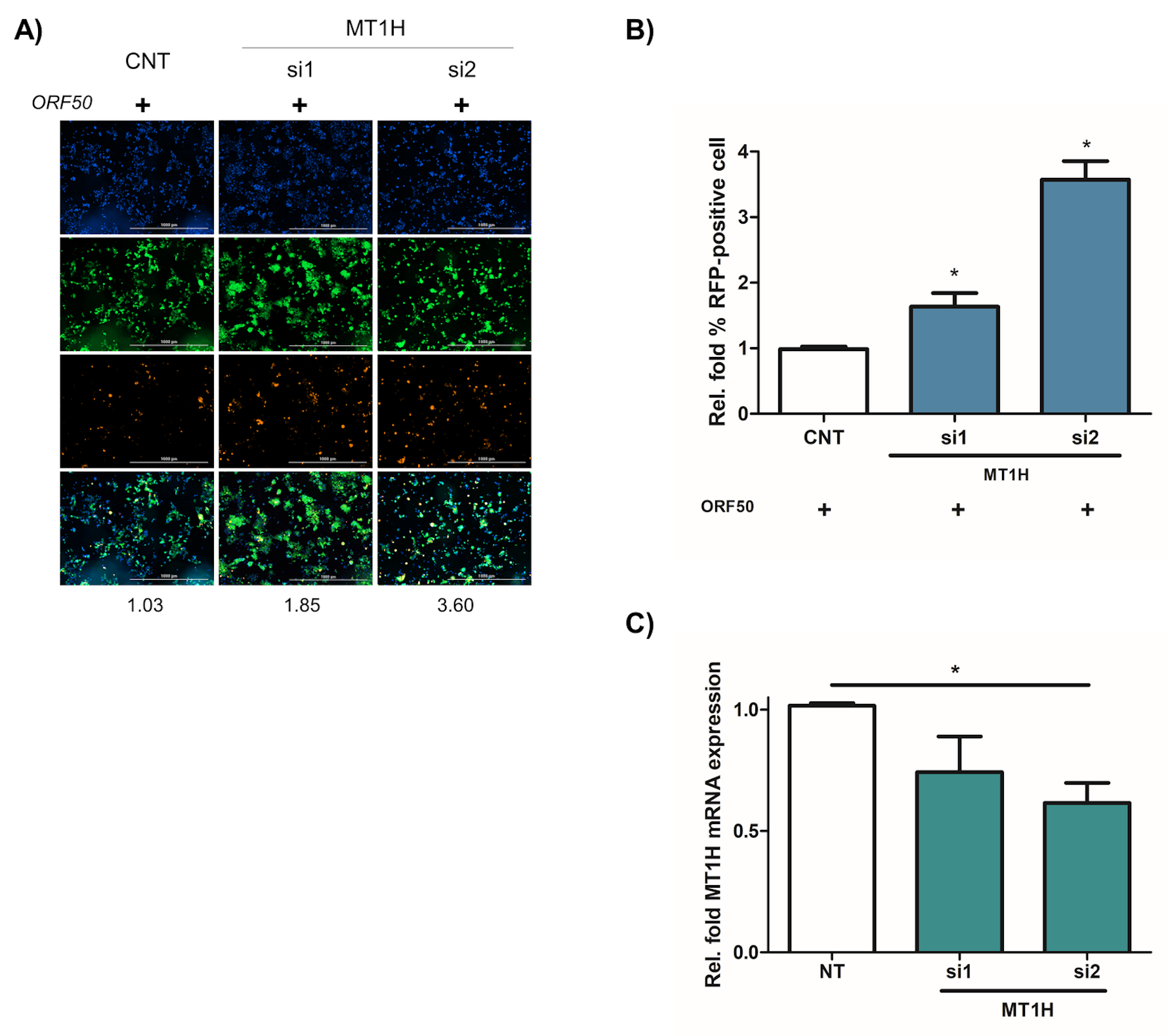

Supplement: S5 Fig — (A) Expression of GFP and RFP proteins in ORF50-transfected HEK293.r219 cells treated with MT1H siRNAs (si1, si2) or control siRNA (CNT) was visualized by fluorescence microscopy (scale bar = 1mm). With GFP at the downstream of eF1α promoter and RFP gene downstream of KSHV lytic PAN promoter, expression of these fluorescent protein indicates KSHV latent infection and lytic reactivation respectively. (B) Percentage of RFP-positive cells undergoing KSHV lytic reactivation was calculated and normalized to the control siRNA (CNT) (n = 3). Results are presented as mean ± SEM (* p<0.05; ** p<0.01; *** p<0.001, two-tailed paired Student t-test). (C) Knockdown efficiency of MT1H siRNAs (si1, si2) in HEK293.r219 cells was analyzed by RT-qPCR at 3 days post of transfection. Results are based on n = 3 independent repeats and presented as mean ± SEM (* p<0.05, two-tailed paired Student t-test). (TIF) [file ppat.1008778.s005.tif]

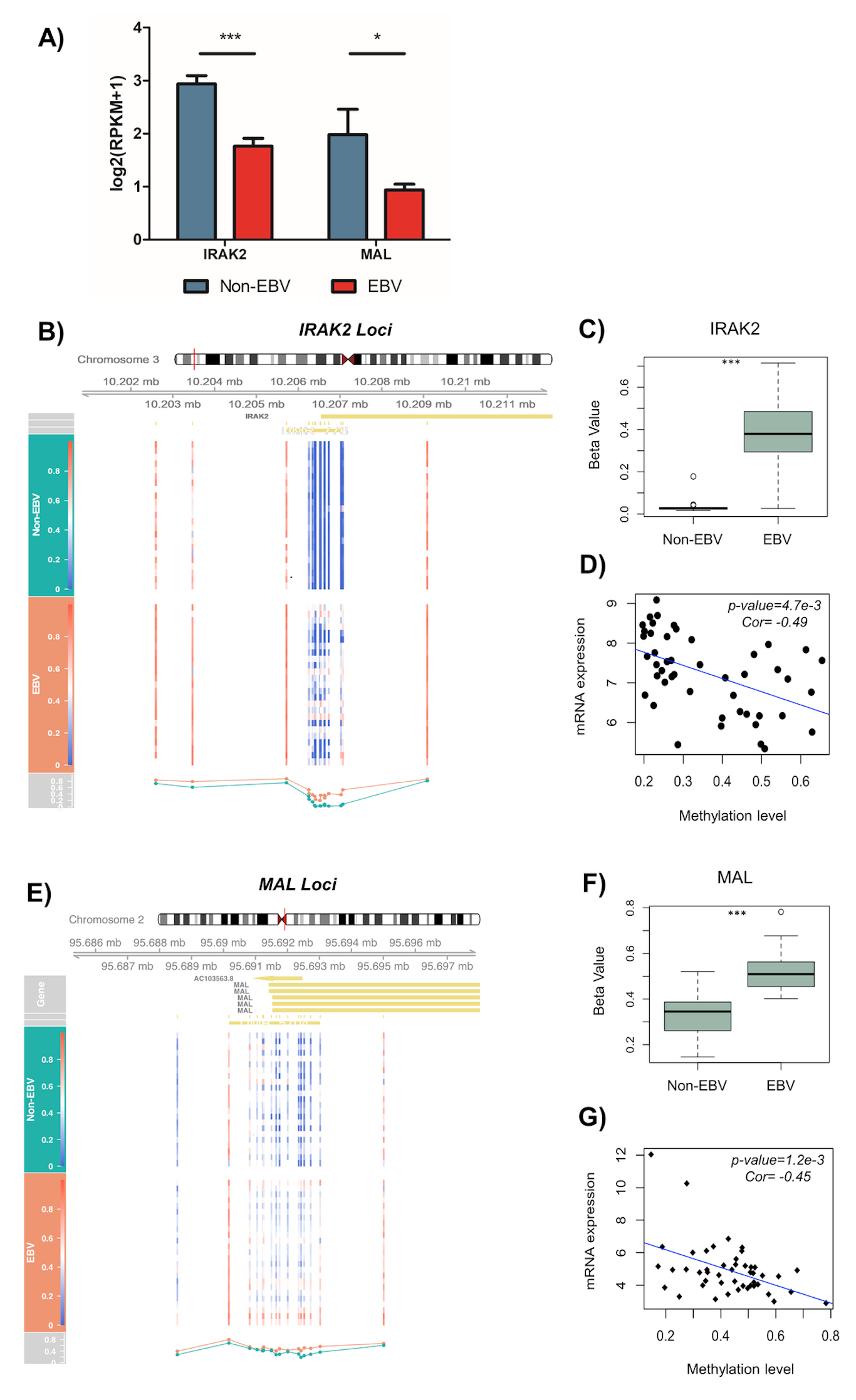

Supplement: S6 Fig — (A) Bar plot showed the normalized mRNA expression for IRAK2 and MAL in EBVaGC vs non-EBV GC. (B, E) Heat map showed the beta value of each CpG probe located within IRAK2 (B) and MAL (E). EBV-negative GC samples (n = 25), upper panel; EBVaGC samples (n = 23), lower panel. (C, F) Box plot showed the distribution of beta values for all CpG probes located within IRAK2 (C) and MAL (F) in EBVaGC and non-EBV GC samples. Results were based on n = 23 (EBV +) and 25 (EBV -) samples and presented as mean ± S.D. (* p<0.05; ** p<0.01; *** p<0.001, Wilcoxon signed-rank test). (D, G) Correlation plot showed the inverse relationship of mRNA expression and methylation beta values for IRAK2 (D) and MAL (G). P-value and Pearson’s correlation coefficient are indicated. (TIF) [file ppat.1008778.s006.tif]

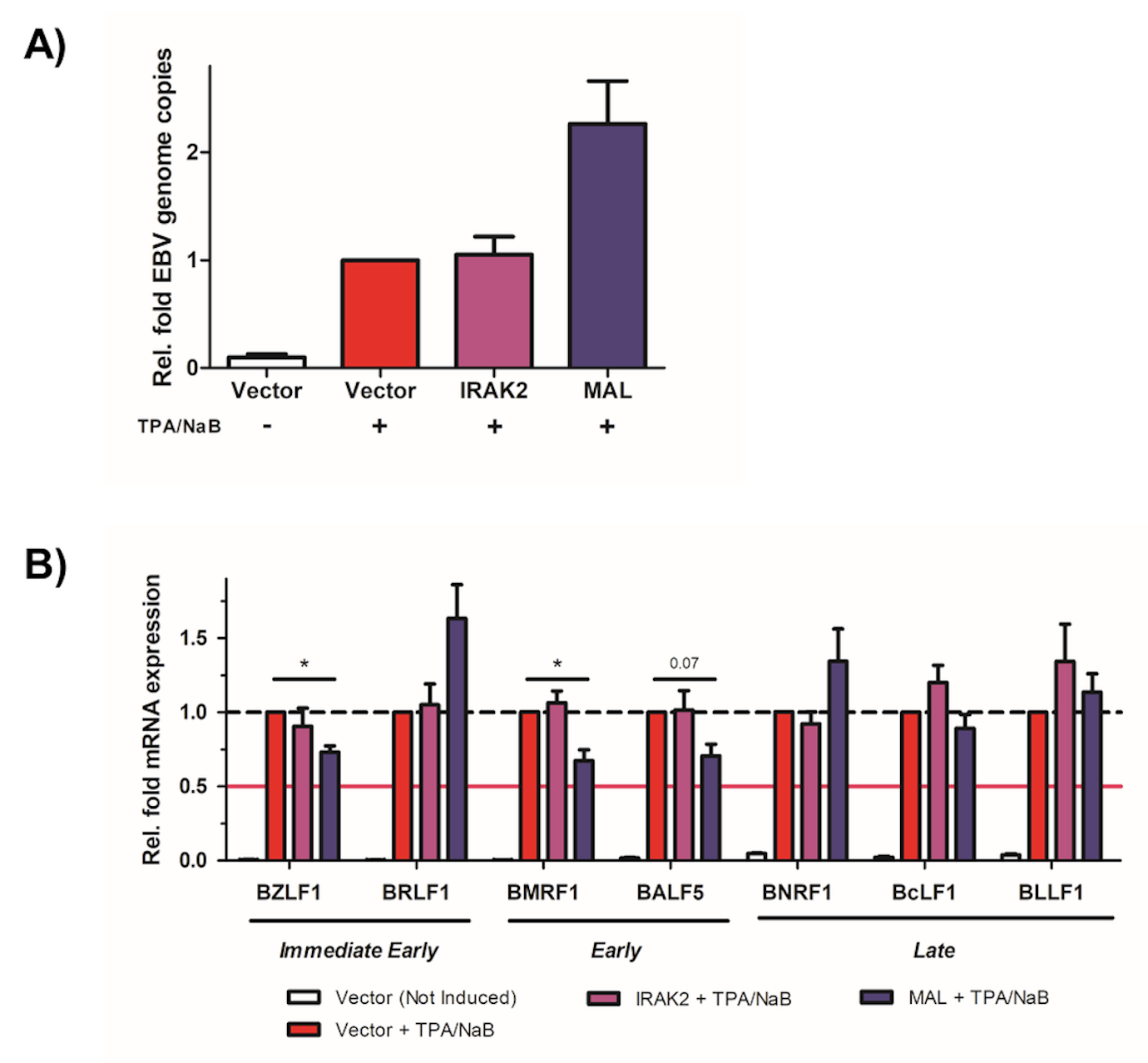

Supplement: S7 Fig — (A) EBV genome copies (EBNA1 DNAs) in TPA/NaB treated AGS-BX cells stably expressing V5-IRAK2, V5-MAL, or the vector control, were measured by qPCR assays at 24 hpi. The relative level of EBNA1 DNAs in TPA/NaB treated AGS-BX cells transduced with the vector control was set up as 1. (B) Expression of EBV lytic genes (BZLF1, BRLF1, BMRF1, BALF5, BNRF1, BcLF1, BLLF1) in TPA/NaB treated or untreated AGS-BX cells stably expressing V5-IRAK2, V5-MAL, or the vector control, were measured by RT-qPCR assays at 24hpi. Results are based on n = 3 independent repeats and presented as mean ± SEM (* p<0.05, two-tailed paired Student t-test). (TIF) [file ppat.1008778.s007.tif]
